# Haploflow: Strain-resolved *de novo* assembly of viral genomes

**DOI:** 10.1101/2021.01.25.428049

**Published:** 2021-01-26

**Authors:** A. Fritz, A. Bremges, Z.-L. Deng, T.-R. Lesker, J. Götting, T. Ganzenmüller, A. Sczyrba, A. Dilthey, F. Klawonn, A.C. McHardy

**Affiliations:** 1 BIFO, Department of Computational Biology, Helmholtz Centre for Infection Research, Braunschweig, Germany; 2 DZIF, German Centre for Infection Research; 3 Institute of Virology, Hannover Medical School, Hannover, Germany; 4 Institute for Medical Virology, University Hospital Tuebingen, Tuebingen, Germany; 5 Faculty of Technology and Center for Biotechnology, Bielefeld University, Bielefeld, Germany; 6 Institute of Medical Microbiology and Hospital Hygiene, University Hospital, Heinrich-Heine-University Düsseldorf, Düsseldorf, Germany; 7 Genome Informatics Section, Computational and Statistical Genomics Branch, National Human Genome Research Institute, Bethesda, MD, 20892, USA; 8 Department of Computer Science, Ostfalia University of Applied Sciences, Wolfenbuettel, Germany; 9 Biostatistics Group, Helmholtz Centre for Infection Research, Braunschweig, Germany

## Abstract

In viral infections often multiple related viral strains are present, due to coinfection or within-host evolution. We describe Haploflow, a de Bruijn graph-based assembler for *de novo* genome assembly of viral strains from mixed sequence samples using a novel flow algorithm. We assessed Haploflow across multiple benchmark data sets of increasing complexity, showing that Haploflow is faster and more accurate than viral haplotype assemblers and generic metagenome assemblers not aiming to reconstruct strains. Haplotype reconstructed high-quality strain-resolved assemblies from clinical HCMV samples and SARS-CoV-2 genomes from wastewater metagenomes identical to genomes from clinical isolates.

Due to co-infection or within host evolution, in viral infections closely related strains, or haplotypes, might be present, with high average nucleotide identity (ANI)^1^ to one another^2–5^. Modern sequencing technologies can capture this variation and computational assembly techniques reconstruct the individual genomes from the resulting data. Currently there are predominantly two types of methods for this problem, viral haplotype assemblers^6,7^ and general (meta)genome assemblers^8–12^. Assembly of individual strains is very difficult, especially if variation is low and few reads span varying sites, resulting in highly fragmented strain genome reconstructions or consensus assemblies^13,14^.

(Meta)genome assemblers usually represent read data initially as a deBruijn (kmer) graph and haplotype assemblers use string graphs^15–18^. String graphs, while being computationally more expensive to construct^19^, due to overlap calculation for all read pairs, have the advantage of detecting mutations that co-occur on a single read^6^, while for deBruijn graphs, this is limited to mutations occurring within the specified *k*-mer length^20^. String graphs are thus more sensitive in matching mutations to strains. If the strains have long stretches of identical sequences, co-occurrences may not happen, which typically is then solved by returning fragmented genome assemblies, where contigs are split between consecutive mutations that cannot be assigned to individual strains. As more contextual information is lost in the deBruijn graph, mutations appear as “bubbles” in the graph, where consecutive vertices are connected by more than one edge^21,22^. (Meta)genome assemblers typically consider these bubbles as errors and follow different approaches for their resolution^22^. The popular SPAdes assembler only considers one path of the bubble, and thus loses the information of the second strain and reconstructs the dominant strain^9,13,23^. MEGAHIT instead terminates contigs prematurely if a bubble is encountered^8^. This leads to fragmented assemblies in the presence of closely related strains^13^.

We here describe Haploflow, a new method and software for the de novo, strain-resolved assembly of viral genomes, which overcomes the problems for both types of methods, i.e. low speed versus loss of strain-specific information, by using information on differential coverage between strains to deconvolute the assembly graph into strain resolved genome assemblies. Haploflow thus does not require reads spanning multiple variable sites for strain resolved assembly of low divergent haplotype populations. As it is based on deBruijn graphs it approaches the runtime behaviour of modern metagenome assemblers. We demonstrate the ability of Haploflow to resolve strains fast and accurately on multiple data sets, including a low complexity HIV strain mixture to a complex, simulated virome sample consisting of 572 viruses with substantial strain-level variation, varying abundances and genome sizes as well as two data sets of clinical human cytomegalovirus (HCMV) and SARS-CoV-2 data.

## Results

We next describe the algorithm for creating and manipulating the assembly graph and the flow algorithm that gave Haploflow its name.

### deBruijn and unitig graph creation

The input to Haploflow is a sequence file including read sequences and specifying the k-mer size for constructing the deBruijn graph. Optionally, the lowest expected strain abundance (or *error rate*) can be specified, leading to removal of more rare kmers from the graph, for graph simplification. Setting the *error-rate* size too low possibly makes the unitig graph and subsequent assembly more complex, while a too high value will prevent low abundant strains from being assembled.

First, a deBruijn graph^21^ is created from the reads, using ntHash^24^ for kmer hashing. Given the reads *R* = {*r*_1_, …, *r*_*n*_}, the deBruijn graph *G* = (*V*, *E*, *k*) contains all substrings of length *k* of *R* as vertices *V* and two vertices *u* and *v* are connected with an edge if the prefix of *u* overlaps with the suffix of length *k* − 1 of *v* or vice versa^25^, i.e. (*u*, *v*) ∈ *E* ⇔ *u*_1…*k*−1_ = *v*_2…*k*_ ⋁ *u*_2…*k*_ = *v*_1…*k*−1_. In addition, *k-*mer counts for every encountered kmer are stored and all weakly connected components (called CCs, a set of vertices that are connected directly or indirectly to each other in the graph) of the graph are calculated. The connected components are found with repeated depth-first searches, until every vertex has been visited and its connected component set. Afterwards, CCs are transformed individually into condensed versions of deBruijn graphs, so-called unitig graphs, where linear paths of vertices, having only one ingoing and one outgoing edge, are collapsed into one vertex.

This unitig graph has the following properties:

- Every remaining vertex is a junction, having more than one ingoing or outgoing edge or being a source or sink. This means that all variation is found in vertices, all non-unique sequences (i.e. occurring in multiple haplotypes) are found in edges.
- The unitig graph is a homeomorphic image of the input deBruijn graph, disregarding error correction. This means that no information is lost and the original deBruijn graph could be reconstructed.

When constructing this unitig graph, for each connected component, so-called junctions, vertices having a different in- from out-degree, or an in- or out-degree of more than one in the de Bruijn graph are identified with a depth-first search. These will be the vertices of the new unitig graph, and their kmers are maintained (Supplementary Fig. S1). The sequence of all the traversed kmers is added to the connecting edge and we define the length of an edge as the length of this sequence in base pairs. Starting from any junction, the next junction in the deBruijn graph is searched, passing vertices with exactly one ingoing and one outgoing edge until the next junction is found. Since all junctions are guaranteed to be searched and the transformation is deterministic, the choice of starting junction does not matter. When the next junction is found, the coverage of all the traversed edges is averaged and checked versus a threshold based on the *error rate* (Supplementary Fig. S2). If it is above, the target junction is also added as a vertex to the unitig graph and an edge with the (averaged) coverage value as the edges coverage is added between the two vertices. If the coverage is below the threshold, then neither the target vertex nor the edge are created and the next outgoing edge of the source is considered. This is repeated until all junctions have been searched, such that no vertices with in-degree = out-degree = 1 are remaining (Figure S1). The resulting unitig graph is usually of drastically reduced size in comparison to the original graph, with sometimes less than 0.01% of vertices remaining. All linear paths of the original graph are condensed into single edges that represent stretches of unique contig sequences.

For every unitig graph a kmer coverage histogram is built (Fig. S2). These histograms reveal several key properties on our data sets: First, the coverage of reads belonging to one genome is approximately normally distributed around the “real” coverage of that genome^19,20^ If multiple sufficiently distinct (in terms of average nucleotide identity) genomes are present in a single unitig graph, then all of them will have a corresponding peak in the histogram. The longer a genome, the more different kmers it includes, and accordingly, the higher the peak. If genomes are very closely related, then the peaks will consist of *k*-mers that are unique to the individual strains and there will be another peak for the common *k*-mers.

Haploflow uses these coverage histograms as indication of the putative number of genomes^26^ and their size relation as well as for error correction. Every read error will create *k* erroneous kmer vertices in the deBruijn graph^27,22^, with low coverage in comparison to the real coverage *cov* of the genomes. Since sequencing errors are rare in Illumina reads, most erroneous kmers will only appear once^28,29^, with fewer kmers appearing multiple times, creating an exponentially decreasing curve in the kmer histogram. This information is factored into the error correction with too rare *k*-mers being removed (red line, Figure S2). The exact method and values used for error correction can be customized by the user, but by default, all *k*-mers with a coverage less than the first inflection point of the coverage histogram are filtered and every *k*-mer which has less than 2% of the coverage of its neighbouring *k*-mers. This parameter can be increased when dealing with long read data to reflect the higher number of errors in current long read technologies.

### Assembly using the flow algorithm

In the second stage the algorithm operates on the unitig graph. It infers and returns a set of contigs based on paths of similar coverages throughout the graph. The flow algorithm consists of three steps that are repeated until the whole graph has been resolved into contigs: (i) finding paths through the graph, (ii) assigning flow values to them, and (iii) determining the path sequence.

**Figure 1:**
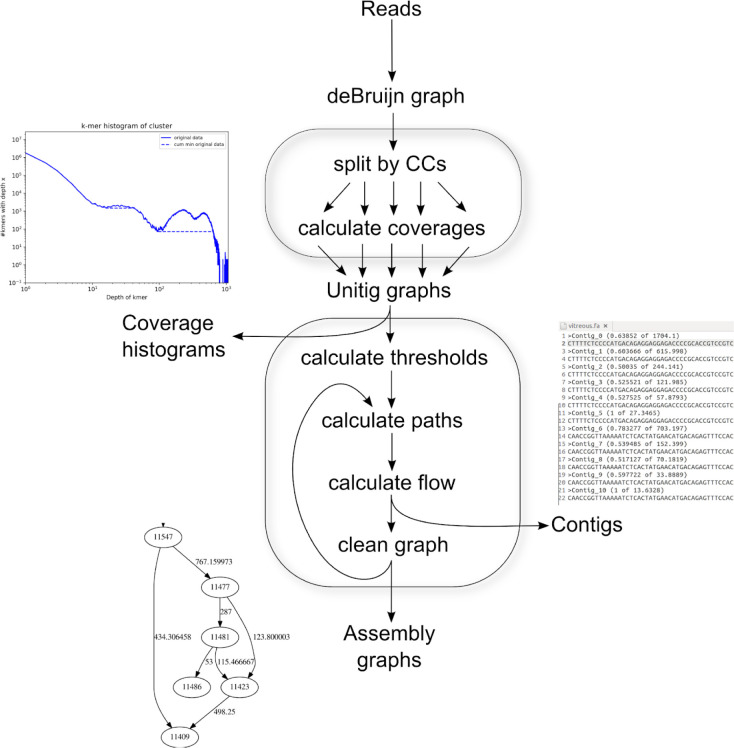
Flow chart of the Haploflow algorithm and its two parts: First the construction of the deBruijn graph and operations thereon, namely splitting it by connected components and calculating coverages. Then the creation of the unitig graphs per CC and the assembly process consisting of calculating the thresholds and the coverage histograms and the putative paths through the graphs. Next is the calculation of the concrete flows and thereby the generation of the contigs and finally the cleaning of the graph and the generation of the assembly graphs. As intermediate output the assembly graph is created during every step (bottom left).

In the first step, the source vertex (with an in-degree of 0) with the highest coverage is selected from the unitig graph. Starting from this source, a modified Dijkstra’s algorithm^30^ is applied, which identifies the fattest path from a source to sink (a vertex having an out-degree of 0) based on edge coverages (Alg. 1, Fig. 1). The fatness of a path is defined by the minimal fatness of the edges on the path. The fatness of an edge is determined as the minimum of its coverage and the fatness of the path from the source until the current edge^31^ and can also be called the “capacity” of the edge. The fattest path from a source to a sink is then determined by following the edges maximising fatness until the sink is found. All edges on this path are then marked with a path number. Subsequently, the coverage for all edges on this path are reduced by the path fatness, the next source is selected and the previous steps are repeated until no edges with coverage remain.

Likely due to technical issues, such as amplification biases^32^ and read errors^33^, and biological structures such as genomic repeats^34^, coverages do not follow a normal distribution globally and consequently some consecutive edges in the assembly graph may exhibit steep changes in coverage. This is the reason why Haploflow uses a two-step procedure for path finding: First, paths are found through the graph as described before. But instead of directly returning contigs for these paths, these paths are only putative, meaning that all paths and changes to the graph are temporary first.

The algorithm of Haploflow is then able to handle heterogeneous coverages across genomes, e.g. highly pronounced in amplicon data or sequence data with high error rates, by using the local, not global coverage distribution, and not absolute coverage, but relative coverage, i.e. the only assumption is that the ratio between haplotypes is somewhat conserved. Additionally, putative paths can get removed, if too many of its edges are already part of a previous putative path (Supplementary Methods). If a path consists almost only of edges that have been used before, it is an indicator that these paths would lead to duplicated contigs. Finally, this results in a graph where all edges are marked with one or more paths they are assumed to be on.


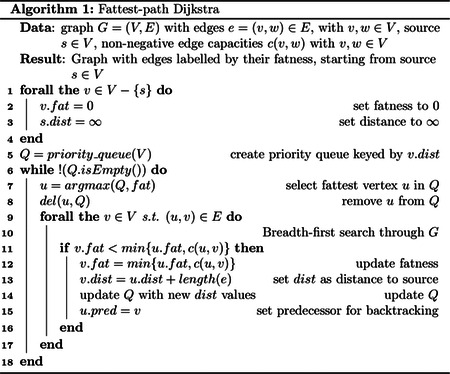


**Alg. 1:** The adapted Dijkstra algorithm used in Haploflow to find fattest paths through the unitig graph. Instead of determining the shortest paths from the source to all vertices, this algorithm determines the fattest path. The fatness is initialised as 0 for all vertices but the source and then the graph is searched using a breadth-first search and based on the fact that the fattest path from a source *s* to a sink *t* is based on the edge with the lowest coverage along this path (lines 9 to 12).

In the second part of the path finding we start again from the source with the highest coverage. Since we have all edges marked with the path that they are on, we can select the edge on the same path which is farthest away from our source and calculate the fattest path from the source to this sink. If Haploflow is not able to resolve the fatness unambiguously, for example because two outgoing edges have almost the same fatness, then the path is terminated in this vertex. This is to prevent formation of chimeric contigs, because locally two strains might have similar coverages. For the final path, a corresponding contig is returned and the coverage reduced permanently (Supplementary Methods). Then all edges with capacity 0 and all vertices without any edges are removed and the flow algorithm started anew from the source vertex. This procedure is repeated until the graph does not have any edges remaining.

Haploflow has multiple parameters that can be set to improve the assembly, if more information is given. If no additional information is given, Haploflow has default settings that usually already provide high quality assemblies. All the evaluations in this article were performed using these default parameters, i.e. a value for *k* of 41, and an *error-rate* of 0.02. The value of *k = 41* was chosen since too small (in comparison to read lengths) values for *k* lead to more ambiguities and a higher *k* might lead to fragmented assemblies. If *k* does not exceed 50% of read-size, the assemblies are of comparable quality. The error-rate parameter was set to 0.02, because this is the value assumed to be the upper bound of errors in short-read sequencing^35^ and can be increased when dealing with more error-prone reads like those from PacBio or Oxford Nanopore.

Additional parameters include a setting for detecting strains with very low absolute abundance (*strict*), for data sets with exactly two strains (*two-strain*), as well as an experimental mode for highly complex data sets with clusters containing five or more closely related strains.

### SARS-CoV-2 clinical and wastewater metagenome data

**Figure 2:**
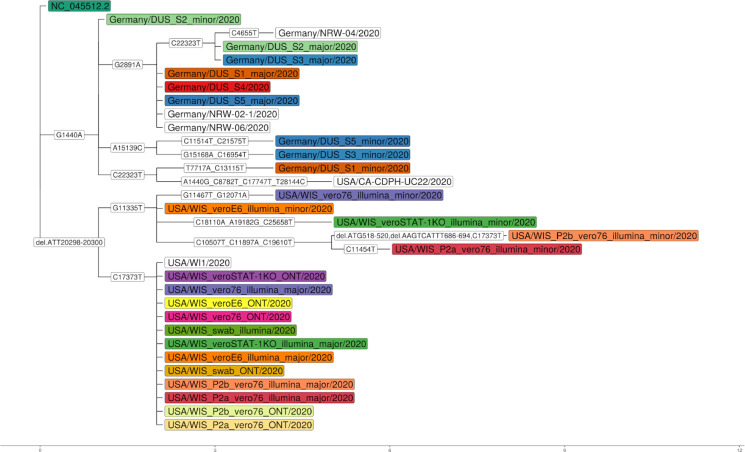
Phylogenetic relationships of reconstructed strain genomes inferred with Raxml^54,55^, including closely related (ANI greater than 99.99%, determined with MASH^56^) strains from GISAID^57^. Strains from the same sample are indicated by color, and “major” and “minor”, based on their inferred abundances. Evolutionary events, including mutations and indels are shown on edges.

We reconstructed viral haplotypes using Haploflow from 17 clinical SARS-CoV-2 samples sampled in Northrhine-Westphalia, Germany (DUS, 5 Illumina short-read samples) and Madison, Wisconsin (WIS, 6 Illumina short-read and 6 Oxford Nanopore long-read samples). After correcting for PCR amplification and sequencing errors (Supplementary Methods), Haploflow identified two strains in nine samples, consistent with in-sample variation^36–38^. The assembled contigs were assessed with QUAST^39^ using the Wuhan-Hu-1 isolate strain (RefSeq NC_045512.2) as reference genome. For all samples, Haploflow produced contigs spanning the complete genome, in 13 cases as a single contig. Haploflow reconstructed the consensus genome sequence(s) found in GISAID^40^ with almost 100% identities as the major strain - from both Illumina and MinION data generated for all WIS samples (Fig. 2). For the Wisconsin strains, which were passaged for up to two rounds in cell cultures, the reconstructed minor strains from short read data had more evolutionary divergences. In comparison to calls from the variant caller Lofreq^41^, (Supplementary Methods), which performs particularly well on mixed strain viral data^14^, both identified 17 (65.4%) of overall 26 identified variant sites (mutations and up to 2bp indels). Interestingly, most of these are C->T transitions, indicating a tendency to alter genome composition^42^ (Supplementary Table S6). In addition, Haploflow identified three longer deletions. Five (19.2%) “unique” LoFreq variants are located in error-prone regions (homopolymeric or strand biased) or at the very end of the genome. Four further low frequency sites (<5%, 15.4%) were found by Haploflow and were also among low frequency Lofreq predictions.

In a study of eight shotgun metagenome samples of sewage from the San Francisco Bay Area^43^, the authors manually assembled consensus SARS-CoV-2 genomes from seven samples and subsequently called variants with inStrain^44^. A comparison to common variants of clinical isolate genomes showed that most of the SNPs found in the data set could be detected in the isolate genomes, with the more (>10%) abundant ones found in strains from California or the US. This and the abundance distribution of some SNPs over time suggested that the data set captured real genomic variation and that different SARS-CoV-2 strains were present in this data set. Haploflow with the option *strict 1* (reduced error correction threshold to account for shallow sequencing depth) and scaffolding (Supplement), assembled full-length SARS-CoV-2 genomes for the same seven samples, recovering two strains for six of them (Supplementary Table S8). Strikingly, for all assemblies identical genomes of clinical SARS-CoV-2 isolates were identified in the GISAID database using minimap^45^ v2.17 (Supplementary Table S8), mostly from samples obtained in the U.S. (5), and California (3), highlighting the ability of Haploflow to recover high quality, strain-resolved viral haplotype genomes from metagenomic data.

### Performance evaluation

We evaluated Haploflow on three simulated data sets with increasing complexity: a mixture of three HIV strains represented by error-free simulated reads, multiple in-vitro created mixtures with different proportions of two HCMV strains sequenced with Illumina HiSeq^46^, and a simulated virome^47,48^ data set of 572 viruses, with 417 genomes in unique taxa and 155 genomes in common strain taxa with up to eleven closely related strains, to assess Haploflow’s ability to assemble complex, larger data sets. Finally, we assembled HCMV genome data from clinical samples collected longitudinally over time from different patients^49^, to characterize the within- and across patient genomic diversity of viral strains, including also larger genomic differences between individual strains in mixed-strain infections, which has not been possible so far. The evaluation was performed using metaQUAST^50^ v.5.0.2, which is commonly used to evaluate metagenome assemblies and provides useful metrics for measuring completeness (genome fraction), continuity (NGA50, largest alignment) and accuracy (mismatches per 100kb, duplication ratio) of assemblies and has specific options for analyzing strain-resolved assemblies. In addition, we calculated metrics for assessing strain-resolved assembly; the strain recall, specifying the fraction of correctly assembled strains (more than 90 (80)% genome fraction and less than 1 (5) mismatches/kb), the strain precision, specifying the fraction of correctly assembled strain genomes of all provided genome assemblies (true positives defined as in recall; total number of genome assemblies estimated as number of ground truth genomes with at least one mapping contig * duplication ratio), as well as the composite assembly quality score, we previously defined^14^. This composite score takes six common assembly metrics (genome fraction, largest alignment, duplication ratio, mismatches per 100 kb, number of contigs and NGA50), normalises them in the range of all results, such that $\text{score}\left(\text{method}\right)\text{ =}\frac{\text{value}\left(\text{method}\right)-\text{min}\left(\text{value}\left(m\in \text{methods}\right)\right)}{\text{max}\left(\text{value}\left(m\in \text{methods}\right)\right)-\text{min}\left(\text{value}\left(m\in \text{methods}\right)\right)}$ for genome fraction, largest alignment and NGA50 and $\text{score}\left(\text{method}\right)\text{ =}\frac{\text{max}\left(\text{value}\left(m\in \text{methods}\right)\right)-\text{value}\left(\text{method}\right)}{\text{max}\left(\text{value}\left(m\in \text{methods}\right)\right)-\text{min}\left(\text{value}\left(m\in \text{methods}\right)\right)}$ for the other metrics and then weighs with a weight of 0.3 for genome fraction and largest alignment, respectively and a weight of 0.1 for the other metrics.

### HIV-3 in silico mixture

HIV, the human immunodeficiency virus, is a single-stranded RNA virus with an approximately 9.5 kb genome that infects humans, causing AIDS (acquired immunodeficiency syndrome). HIV evolves rapidly within the host and may also present as multi-strain infections^51,52^. The three HIV-1 strains 89.6, HXB2 and JR-CSF, which are commonly used to evaluate viral haplotype assemblers^53,54^, were downloaded from NCBI RefSeq^55^, mixed in the proportions 10:5:2 and error-free reads with a length of 150bp and depth of 20,000 created with CAMISIM^56^ and the wgsim read simulator^57^. These genomes differ mainly by SNPs and have an average nucleotide identity (ANI) of ~95%. This threshold was chosen, because experiments on MEGAHIT and metaSPAdes showed that genomes more closely related than 95% will not be resolved^56^.

We benchmarked the quality of strain-resolved Haploflow assemblies for the three strain HIV data against five other *de novo* assemblers (SPAdes, metaSPAdes, megahit, PEHaplo, SAVAGE in *de novo* mode) with metaQUAST v.5.0.2, using multiple parameter settings, if defaults settings were undefined (QuasiRecomb, PEHaplo). Furthermore, we assessed five reference-based assemblers (GAEseq^58^, SAVAGE ref-based mode, PredictHaplo, QuasiRecomb and CliqueSNV), which were provided with one strain genome for assembly.

Of all evaluated *de novo* assemblers, Haploflow performed best across all metrics and the composite assembly score (Figure S2), assembling all three strains almost completely (more than 90%), with less than 1 mismatch/kb, providing no false positive strain assemblies - that for some methods (QuasiRecomb) reached several thousand strains - and with more than double the assembly contiguity (NGA50) than the second best method (PEHaplo). Haploflow was the only method assembling all strain genomes into complete contigs. Also in comparison to the reference-based assemblers, Haploflow performed best. SAVAGE in reference-based mode, run on a subsample of the data, performed similarly well in five of the eight metrics, however, provided a substantially more fragmented assembly (lower NGA50, more contigs) and a strain genome with more mismatches. Haploflow also closely estimated the true underlying strain proportions, with predicted coverages of 10,371 for HIV 89.6, 5,372 for HIV HXB2 and 1,745 for HIV JR-CSF.

### HCMV in vitro mixtures

We next evaluated Haploflow on six lab-created mixtures of two HCMV strains sequenced with Illumina MiSeq^59^. HCMV is one of the largest human pathogenic viruses, causing severe illness in immunocompromised patients and infants, and possessing a double stranded DNA genome of more than 220 kb^60^. The data set includes two different strain mixtures, denoted “TA” (strains TB40 and AD169, 97.9% ANI) and “TM” (strains TB40 and Merlin, 97.7% ANI), with three different mixture ratios each (1:1, 1:10 and 1:50), allowing us to test the ability of assemblers to resolve strains at varying abundances. We ran Haploflow on these data and compared the results to those of twelve other assemblers. These include nine (meta)genome assemblers (ABySS, IDBA, MEGAHIT, metaSPAdes, Ray, SPAdes, tadpole, IVA^61^ and Vicuna^62^) also widely used for single-cell and virome data because of their accuracies and speed, and three specialised viral haplotype assemblers delivering a result (reference-based SAVAGE, VirGenA^63^ and PEHaplo). Four more viral haplotype assemblers, SAVAGE *de novo*, PredictHaplo^64^, CliqueSNV^65^ QuasiRecomb^46^, either did not return results^14^ or were terminated after ten days (Haploflow on average required 1.5h per sample). Assemblies were evaluated using metaQUAST v.5.0.2 with the benchmarking workflow QuasiModo^14^, based on common assembly metrics, the composite assembly score, recall and precision in strain-resolved genome assembly, as before, and the top performing methods falling in the 95–100% range of results identified for every metric.

Of the 12 evaluated de novo assemblers, Haploflow scored best in 5 of the 8 metrics, followed by metaSPAdes (best in 2 of 8: NGA50, duplication ratio), while PEHaplo, tadpole, IDBA, Vicuna and IVA each scored best for one metric, respectively (Supplementary Table S2). Haploflow assemblies were of very high quality, recovering the most correct strain genomes (10 of 12), providing the best strain precision and composite assembly score (9.34 of 10), highest genome fraction (83.87%) and the most contiguous assemblies (NGA50 62,560). Interestingly, the similarly good NGA50 values of metaSPAdes and Haploflow were obtained in different ways, for the former due to a more contiguous assembly for the abundant strain, while only Haploflow and the haplotype assembler SAVAGE in reference-mode recovered more than 50% of the low abundant strain in several mixtures.

### Simulated virome data set

To test Haploflows ability to recover viral strain genomes from complex data sets, we evaluated Haploflow, MEGAHIT and metaSPAdes on the simulated virome data set from the Namib desert^47^, which includes short-read data simulated from an *in-silico* mixture of 572 viral genomes created to assess different assemblers^48^. It was not possible to run the *reference-free* haplotype-assemblers (SAVAGE, PEHaplo) on this data set. To assess the evolutionary divergence between the viral genomes, we identified clusters of similar genomes using dRep^68^, which resulted in 469 clusters total, out of which 52 clusters had at least two members with more than 95% ANI (average nucleotide identity), resulting in 417 “unique” genomes and 155 genomes in common strain clusters. The 95% threshold was chosen since MEGAHIT and metaSPAdes are only able to resolve genomes less similar than that^56^.

For the 155 common strain genomes, Haploflow correctly assembled 13–28.6% more sequence (62.85% genome fraction versus 55.58% and 48.88% for SPAdes and MEGAHIT, respectively). This was even more pronounced for clusters with genomes of at least eight-fold coverage, for which 19.8–37.5% more genome sequence was correctly assembled (89.37% versus 74.58% and 64.99% for SPAdes and MEGAHIT, respectively). For the less abundant strains from these clusters, 32.7–45.3% more genome sequence was correctly assembled (87.37% versus 65.85% and 60.12% genome fraction, respectively). Even for the complete data set with “unique” genomes and low abundant genomes, Haploflow reconstructed genome fractions similar to the MEGAHIT and metaSPAdes assemblers (72.2% and 68.6% versus 66.6% genome fraction; Table S5), which performed best in the original publication.

### Analysis of clinical HCMV data

We used Haploflow with default parameters to reconstruct genomes from longitudinal clinical samples of eight HCMV positive patients, who had multi-strain infections^59^ (Supplementary Table S5). QUAST was used to map HaploFlow’s contigs against the consensus strain of the first time point as reference genome, as the exact underlying strain genomes in the samples are unknown. Using the QUAST output, in particular the duplication ratio, the number of strains predicted by HaploFlow was determined by rounding the duplication ratio and then clustering the contigs into that many clusters using HaploFlow’s predicted flow (using python’s sklearn^69^ *k-*means method). For each of the clusters, QUAST was re-run, again using the consensus as reference genome. Since the resulting genomes, in particular the low abundant (minor) strains, will inherently be different to the consensus to some degree, only the genome fraction is considered a relevant metric here. Additionally, to confirm that HaploFlow created accurate strain-resolved contigs instead of consensus contigs, we compared clusters from the same patient at different time points with each other, finding that contigs from two clusters from consecutive time points showed ~99.9% ANI, while randomly matched clusters only had ~98% ANI.

For all patients with multi-strain infections, Haploflow reconstructed multiple complete genomes for at least one time point. For most patients, sequencing data of their infection exist for multiple time points and Haploflow recovered all strains, if the abundance of the lower abundant strain exceeded 6.8%, once as low as 6.1% (Supplementary Table S5). Haploflow reconstructed the genomes of both, or in two cases three, strains infecting the patient. Haploflow correctly predicted at least one lower abundant strain for 19 of 23 (82.6%) time points with multiple strain infections, correctly predicted 44 strains of the total 48 strains (91.7% recall) and only predicted (parts of) three unconfirmed, additional strains (93.2% precision). Haploflow also reproduced the results from the original publication^59^, where a strain with a structurally altered genome established itself as dominant over two consecutive time points in patients SCTR1 and SCTR11, and also recovered three distinct strains from the SCTR18 sample (Supplementary Table S5). The samples for which Haploflow did not assemble a second strain had either a very low abundant second strain (4% for SCTR1-day91), a shallow coverage (coverage of 22 and 38 for SCTR1–245days and SCTR3–320days) or a combination therof (6.8% variant at 105 coverage for SCTR1–194days).

Finally, we tested Haploflow on a HCMV sample for which the genotypes and proportion of both strains were known (Supplementary Figure 4). Haploflow reconstructed both strains with a total of 19 contigs, 4 for the high abundant strain and 15 for the low abundant. The high abundant strain assembly matched the consensus strain with 0.87 mismatches/100kb, a NGA50 value of 113,718 and 99.84% genome fraction. The contigs produced for the low abundant strain were also evaluated using the consensus sequence, showing 94.58% genome fraction and an NGA50 of 62,533. The largest contig showed a 7,830 base sequence not present in the consensus sequence, but matching perfectly to another (BE/43/2011) HCMV sequence, demonstrating the ability of Haploflow to accurately phase different haplotypes.

### Runtime and memory consumption

Haploflow’s run time depends on the three main steps (Fig. 1): first reading in the read data and building the deBruijn graph. For this every read is split into *k*-mers, with a time complexity in O(*n*) for the number of reads *n*. Since the maximal number of *k-*mers is constant in the number of reads and the length of the reads with |*k*| = (length(*n*) - *k*) · |*n*|, it is also in O(*k*). Next the graph is split into CCs and the unitig graph constructed, with a time complexity of O(*k*) using Tarjan’s algorithm^70^. Finally the overall complexity of the assembly step is dominated by finding the paths through the unitig graph. While theoretically there is an exponential number of different paths through a graph, every vertex can only be the source of a path once and every path has length at most *k*, since vertices cannot be visited multiple times on the same path. The worst-case complexity of the assembly step is thus in O(*k*^2^), where *k* is the number of distinct *k*-mers. In practice, the number of paths is usually limited by the number of different strains, causing this step to also be linear time complexity.

For runtime assessment we compared Haploflow to SAVAGE and PEHaplo, the only other haplotype assemblers able to process the HCMV data, though SAVAGE only in *reference-based* mode, as well as metaSPAdes and MEGAHIT, which performed closest to Haploflow in terms of the summary score or is a very fast metagenome assembler, respectively (Table 1). On the HIV data, Haploflow was more than twice as fast than SAVAGE. The running time and memory requirements of Haploflow and metaSPAdes were comparable, while MEGAHIT was most efficient.

On the HIV three strain and the HCMV two strain mixtures, building the deBruijn graph and creation of the unitig graphs from the reads dominated the overall running time. For the HIV data, building the deBruijn and unitig graphs took ~8 minutes on a laptop with 4 cores and 16 GB RAM. The resulting single unitig graph included 281 vertices and assembly finished after 0.6 seconds. For the HCMV data, assembly on the same laptop required ~100 minutes, of which 85 were used for building the deBruijn and unitig graphs from the reads.

## Discussion and conclusions

Viral pathogens can evolve rapidly, leading to infections with multiple strains either by within-host evolution or multiple infections of the same host. Reconstructing their genomes in a strain-resolved manner can substantially advance our understanding and capabilities to combat the diseases they cause. It is also key for genomic epidemiology, i.e. tracing viral spread using genomic information^71,72^ and genome-based viral phenotyping^73^. Strains can differ in their phenotypes, such as virulence, resistance, or the degree of their immune resistance to host immunity, which may be critical for the choice of therapy.

Strain-resolved *de novo* assembly from short-read as well as long read data generated in viral genome sequencing, however, is also extremely challenging. Haploflow fills a void between fast metagenome assemblers not aiming for strain-level resolution, and viral haplotype assemblers for small viral genomes of a few kb in size. It combines the best of both worlds for strain-resolved genome assembly, by using the fast algorithms of the metagenome assemblers, i.e. deBruijn graph based assembly, together with a specialised flow algorithm for capturing strain variation, which allows to link variants that do not co-occur on reads.

Taken together, our results demonstrate a substantial performance improvement in strain-resolved assembly for Haploflow in comparison to sixteen other metagenome and viral haplotype assemblers evaluated across different benchmark data sets. The benchmark experiments on data sets with varying numbers of strains and abundances demonstrated that Haploflow can handle data sets with substantial variation in genomic coverage introduced by amplicon sequencing and resolved strains at different degrees of evolutionary divergences well, ranging from 95% ANI (HIV), over 98% ANI (HCMV), to more than 99% ANI (SARS-CoV-2 data). On the six lab-generated HCMV mixed strain data sets, Haploflow was top scoring in the most metrics (5 of 8) in comparison to twelve other assemblers. This performance improvement in strain recall, strain precision, composite score, genome fraction and NGA50 was largely due to a better assembly of the less abundant strains. Except for Haploflow and SAVAGE **no** method assembled low abundant strains to 50% on average and Haploflow had a *far* higher NGA50, creating long contigs rather than a highly fragmented assembly. On the clinical HCMV data tested, Haploflow almost perfectly (91.7% recall and 93.6% precision) assembled strains with variants predicted by variant callers and very closely predicted the abundances of second and third strains. On a three strain HIV data set, Haploflow assembled all three genomes almost entirely, with very few mismatches. This is reflected in Haploflow scoring top in all eight metrics, with a composite assembly score of 9.66 (out of 10), in comparison to 8.02 for the best *reference-based* assembler PredictHaplo, and of 6.28 for the best *reference-free* assembler PEHaplo.

Benchmarking on a rather complex simulated virome data set with 417 taxa with unique genomes and 155 genomes in common strain taxa showed that Haploflow successfully assembled 2–3 strains for “common strain taxa” with 2–11 strains, substantially better so than the state-of-the-art metagenome assemblers able to process these data, that the tested haplotype assemblers could not. This effect was particularly pronounced for strain genome coverages within a favorable (>8) range for assembly. The abundance distribution of taxa in microbial communities is assumed to be oftentimes log-normal^13^, with only a few abundant and a long tail of very low abundant ones with consequently low coverages. This indicates that Haploflow is suitable for processing many real world data sets and characterizing the more abundant strains, similar to the reference-based StrainPhlan strain-typing software^74^. Finally, Haploflow reconstructed multiple, full length SARS-CoV-2 strains from a multi-sample wastewater metagenome data set with exact matches to clinical isolate genomes found in the GISAID database, highlighting the ability of Haploflow to recover high quality, strain-resolved viral haplotype genomes from metagenomic data.

In addition to short-read data, Haploflow also allows processing of long read data, which we demonstrated on the SARS-CoV-2 clinical data sets. For most applications dealing with low viral loads (e.g. the SARS-CoV-2 sequencing demonstrated in this article), PCR amplification is necessary to enrich viral reads. This naturally limits the possible maximum read length to the length of the PCR product, which is for those applications in the domain of short-read sequencing. The speed of the Haploflow algorithm principally also allows its extension to bacterial data, e.g. by adding multi-core and multi-*k* support and modules for handling differently sized and structured microbial genomes. Thus strain-resolved assembly from metagenome data for microbial taxa with several closely related strains could be a future application.

## Supplementary Material

1

## Figures and Tables

**Figure 3:**
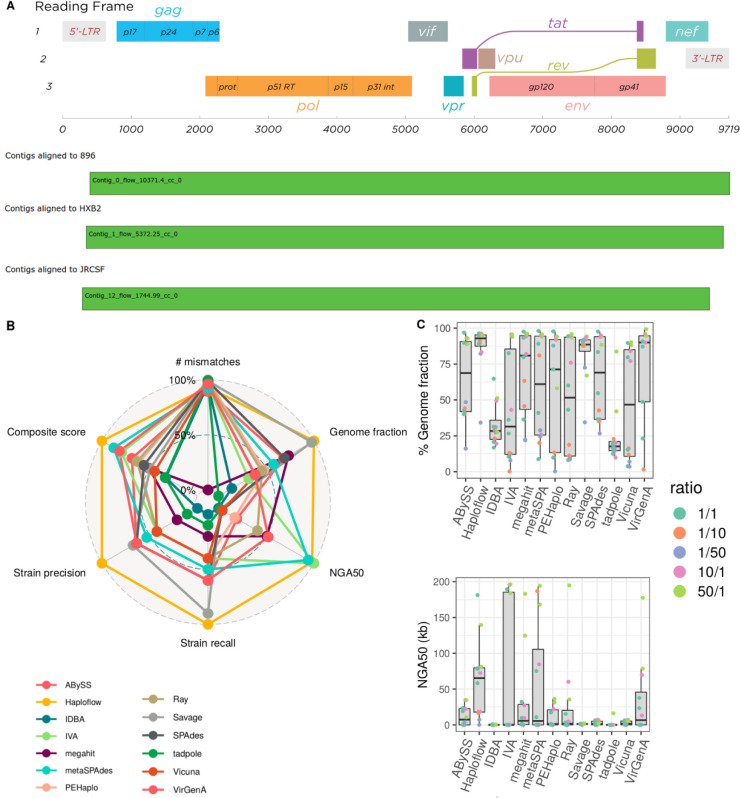
**A:** HI virus genome structure^66^ and Icarus plots^67^ for three HIV strains reconstructed by Haploflow. For each of the three reference genomes there is one contig spanning almost the complete genome. **B:** Radar plot of relative performance with commonly used and strain-resolved genome assembly metrics for Haploflow and 12 other methods on the HCMV benchmark data (best values are at 100%, see Performance evaluation). Haploflow, in orange, ranks first in genome fraction, Strain recall, Strain precision and Composite score. **C:** Boxplots with median and interquartile range of genome fraction and NGA50 values across samples for different methods.

**Table 1:** Run time and memory consumption of Haploflow, SAVAGE in de novo mode (version 0.4.1), metaSPAdes (3.14) and MEGAHIT (1.2.9). Time and memory is averaged for the HCMV mixtures. SAVAGE did not successfully complete on the HCMV in-vitro mixtures and the simulated virome data. Values were calculated using linux’ time command.

Software/ Dataset	HIV 3 in silico mixture		HCMV in vitro mixture		Simulated virome	
Metric	CPU *user* time (seconds)	Memory peak (GB)	Avg. CPU user time (seconds)	Avg. memory peak (GB)	CPU user time (seconds)	Memory peak (GB)
Haploflow	724	**0.009**	5,170	17.509	18,245	47.678
SAVAGE	110,208	102.938	75,518	17.658	**-**	**-**
PEHaplo	10,127	11.819	58,920	13.998	-	-
metaSPAdes	1,500	1.054	42,906	65.641	25,996	23.399
MEGAHIT	**250**	0.269	**2,910**	**0.754**	**9,690**	**2.148**

## References

[1] Richter M. & Rosselló-Móra R. Shifting the genomic gold standard for the prokaryotic species definition. Proc. Natl. Acad. Sci. U. S. A. 106, 19126–19131 (2009). 19855009 10.1073/pnas.0906412106 PMC2776425

[2] Waner J. L. Mixed viral infections: detection and management. Clin. Microbiol. Rev. 7, 143–151 (1994). 8055464 10.1128/cmr.7.2.143 PMC358314

[3] Ghedin E. Mixed Infection and the Genesis of Influenza Virus Diversity. J. Virol. 83, 8832–8841 (2009). 19553313 10.1128/JVI.00773-09 PMC2738154

[4] Ojosnegros S., Beerenwinkel N. & Domingo E. Competition-colonization dynamics: An ecology approach to quasispecies dynamics and virulence evolution in RNA viruses. Commun. Integr. Biol. 3, 333–336 (2010). 20798818 10.4161/cib.3.4.11658 PMC2928310

[5] Kumar N., Sharma S., Barua S., Tripathi B. N. & Rouse B. T. Virological and Immunological Outcomes of Coinfections. Clin. Microbiol. Rev. 31, (2018). 10.1128/CMR.00111-17 PMC6148187 29976554

[6] Baaijens J. A. & Schönhuth A. Overlap graph-based generation of haplotigs for diploids and polyploids. Bioinformatics 35, 4281–4289 (2019). 30994902 10.1093/bioinformatics/btz255

[7] Töpfer A. Viral Quasispecies Assembly via Maximal Clique Enumeration. PLOS Comput. Biol. 10, e1003515 (2014). 24675810 10.1371/journal.pcbi.1003515 PMC3967922

[8] Li D., Liu C.-M., Luo R., Sadakane K. & Lam T.-W. MEGAHIT: an ultra-fast single-node solution for large and complex metagenomics assembly via succinct de Bruijn graph. Bioinforma. Oxf. Engl. 31, 1674–1676 (2015). 10.1093/bioinformatics/btv033 25609793

[9] Nurk S., Meleshko D., Korobeynikov A. & Pevzner P. A. metaSPAdes: a new versatile metagenomic assembler. Genome Res. 27, 824–834 (2017). 28298430 10.1101/gr.213959.116 PMC5411777

[10] Peng Y., Leung H. C. M., Yiu S. M. & Chin F. Y. L. IDBA-UD: a de novo assembler for single-cell and metagenomic sequencing data with highly uneven depth. Bioinforma. Oxf. Engl. 28, 1420–1428 (2012). 10.1093/bioinformatics/bts174 22495754

[11] Boisvert S., Laviolette F. & Corbeil J. Ray: Simultaneous Assembly of Reads from a Mix of High-Throughput Sequencing Technologies. J. Comput. Biol. 17, 1519–1533 (2010). 20958248 10.1089/cmb.2009.0238 PMC3119603

[12] Simpson J. T. ABySS: A parallel assembler for short read sequence data. Genome Res. 19, 1117–1123 (2009). 19251739 10.1101/gr.089532.108 PMC2694472

[13] Sczyrba A. Critical Assessment of Metagenome Interpretation—a benchmark of metagenomics software. Nat. Methods 14, 1063–1071 (2017). 28967888 10.1038/nmeth.4458 PMC5903868

[14] Deng Z.-L. Evaluating assembly and variant calling software for strain-resolved analysis of large DNA-viruses. bioRxiv 2020.05.14.095265 (2020) doi:10.1101/2020.05.14.095265 PMC8138829 34020538

[15] Eriksson N. Viral Population Estimation Using Pyrosequencing. PLoS Comput. Biol. 4, (2008). 10.1371/journal.pcbi.1000074 PMC2323617 18437230

[16] Astrovskaya I. Inferring viral quasispecies spectra from 454 pyrosequencing reads. BMC Bioinformatics 12, S1 (2011). 10.1186/1471-2105-12-S6-S1 PMC3194189 21989211

[17] Mancuso N., Tork B., Skums P., Măndoiu I. & Zelikovsky A. Viral quasispecies reconstruction from amplicon 454 pyrosequencing reads. in 2011 IEEE International Conference on Bioinformatics and Biomedicine Workshops (BIBMW) 94–101 (2011). doi:10.1109/BIBMW.2011.6112360

[18] O’Neil S. T. & Emrich S. J. Haplotype and minimum-chimerism consensus determination using short sequence data. BMC Genomics 13, S4 (2012). 10.1186/1471-2164-13-S2-S4 PMC3394418 22537299

[19] Miller J. R., Koren S. & Sutton G. Assembly Algorithms for Next-Generation Sequencing Data. Genomics 95, 315–327 (2010). 20211242 10.1016/j.ygeno.2010.03.001 PMC2874646

[20] Schatz M. C., Delcher A. L. & Salzberg S. L. Assembly of large genomes using second-generation sequencing. Genome Res. 20, 1165–1173 (2010). 20508146 10.1101/gr.101360.109 PMC2928494

[21] Pevzner P. A., Tang H. & Waterman M. S. An Eulerian path approach to DNA fragment assembly. Proc. Natl. Acad. Sci. U. S. A. 98, 9748–9753 (2001). 11504945 10.1073/pnas.171285098 PMC55524

[22] Pevzner P. A., Tang H. & Tesler G. De Novo Repeat Classification and Fragment Assembly. Genome Res. 14, 1786–1796 (2004). 15342561 10.1101/gr.2395204 PMC515325

[23] Bankevich A. SPAdes: A New Genome Assembly Algorithm and Its Applications to Single-Cell Sequencing. J. Comput. Biol. 19, 455–477 (2012). 22506599 10.1089/cmb.2012.0021 PMC3342519

[24] Mohamadi H., Chu J., Vandervalk B. P. & Birol I. ntHash: recursive nucleotide hashing. Bioinformatics 32, 3492–3494 (2016). 27423894 10.1093/bioinformatics/btw397 PMC5181554

[25] Compeau P. E. C., Pevzner P. A. & Tesler G. Why are de Bruijn graphs useful for genome assembly? Nat. Biotechnol. 29, 987–991 (2011). 22068540 10.1038/nbt.2023 PMC5531759

[26] Chor B., Horn D., Goldman N., Levy Y. & Massingham T. Genomic DNA k-mer spectra: models and modalities. Genome Biol. 10, R108 (2009). 19814784 10.1186/gb-2009-10-10-r108 PMC2784323

[27] Idury R. M. & Waterman M. S. A New Algorithm for DNA Sequence Assembly. J. Comput. Biol. 2, 291–306 (1995). 7497130 10.1089/cmb.1995.2.291

[28] Melsted P. & Halldórsson B. V. KmerStream: streaming algorithms for k-mer abundance estimation. Bioinformatics 30, 3541–3547 (2014). 25355787 10.1093/bioinformatics/btu713

[29] Chikhi R. & Medvedev P. Informed and automated k-mer size selection for genome assembly. Bioinformatics 30, 31–37 (2014). 23732276 10.1093/bioinformatics/btt310

[30] Dijkstra E. W. A note on two problems in connexion with graphs. (1959).

[31] luca. CS 261 Lecture 10: the fattest path. in theory https://lucatrevisan.wordpress.com/2011/02/04/cs-261-lecture-10-the-fattest-path/ (2011).

[32] Aird D. Analyzing and minimizing PCR amplification bias in Illumina sequencing libraries. Genome Biol. 12, R18 (2011). 21338519 10.1186/gb-2011-12-2-r18 PMC3188800

[33] Schirmer M., D’Amore R., Ijaz U. Z., Hall N. & Quince C. Illumina error profiles: resolving fine-scale variation in metagenomic sequencing data. BMC Bioinformatics 17, 125 (2016). 26968756 10.1186/s12859-016-0976-y PMC4787001

[34] Sivadasan N., Srinivasan R. & Goyal K. Kmerlight: fast and accurate k-mer abundance estimation. ArXiv160905626 Cs (2016).

[35] Laehnemann D., Borkhardt A. & McHardy A. C. Denoising DNA deep sequencing data—high-throughput sequencing errors and their correction. Brief. Bioinform. 17, 154–179 (2016). 26026159 10.1093/bib/bbv029 PMC4719071

[36] Walker A. Genetic structure of SARS-CoV-2 in Western Germany reflects clonal superspreading and multiple independent introduction events. medRxiv 2020.04.25.20079517 (2020) doi:10.1101/2020.04.25.20079517 PMC7336109 32524946

[37] Rose R. Intra-host site-specific polymorphisms of SARS-CoV-2 is consistent across multiple samples and methodologies. medRxiv 2020.04.24.20078691 (2020) doi:10.1101/2020.04.24.20078691

[38] Moreno G. K. Limited SARS-CoV-2 diversity within hosts and following passage in cell culture. bioRxiv 2020.04.20.051011 (2020) doi:10.1101/2020.04.20.051011

[39] Gurevich A., Saveliev V., Vyahhi N. & Tesler G. QUAST: quality assessment tool for genome assemblies. Bioinforma. Oxf. Engl. 29, 1072–1075 (2013). 10.1093/bioinformatics/btt086 PMC3624806 23422339

[40] Shu Y. & McCauley J. GISAID: Global initiative on sharing all influenza data – from vision to reality. Eurosurveillance 22, (2017). 10.2807/1560-7917.ES.2017.22.13.30494 PMC5388101 28382917

[41] Wilm A. LoFreq: a sequence-quality aware, ultra-sensitive variant caller for uncovering cell-population heterogeneity from high-throughput sequencing datasets. Nucleic Acids Res. 40, 11189–11201 (2012). 23066108 10.1093/nar/gks918 PMC3526318

[42] Holmes E. C. The Evolution and Emergence of RNA Viruses.(Oxford University Press, 2009).

[43] Crits-Christoph A. Genome sequencing of sewage detects regionally prevalent SARS-CoV-2 variants. medRxiv 2020.09.13.20193805 (2020) doi:10.1101/2020.09.13.20193805 PMC7845645 33468686

[44] Olm M. R. InStrain enables population genomic analysis from metagenomic data and rigorous detection of identical microbial strains.http://biorxiv.org/lookup/doi/10.1101/2020.01.22.915579 (2020) doi:10.1101/2020.01.22.915579

[45] Li H. Minimap and miniasm: fast mapping and de novo assembly for noisy long sequences. Bioinformatics 32, 2103–2110 (2016). 27153593 10.1093/bioinformatics/btw152 PMC4937194

[46] Caporaso J. G. Ultra-high-throughput microbial community analysis on the Illumina HiSeq and MiSeq platforms. ISME J. 6, 1621–1624 (2012). 22402401 10.1038/ismej.2012.8 PMC3400413

[47] Hesse U. Virome Assembly and Annotation: A Surprise in the Namib Desert. Front. Microbiol. 8, 13 (2017). 28167933 10.3389/fmicb.2017.00013 PMC5253355

[48] Sutton T. D. S., Clooney A. G., Ryan F. J., Ross R. P. & Hill C. Choice of assembly software has a critical impact on virome characterisation. Microbiome 7, 12 (2019). 30691529 10.1186/s40168-019-0626-5 PMC6350398

[49] Hage E. Characterization of Human Cytomegalovirus Genome Diversity in Immunocompromised Hosts by Whole-Genome Sequencing Directly From Clinical Specimens. J. Infect. Dis. 215, 1673–1683 (2017). 28368496 10.1093/infdis/jix157

[50] Mikheenko A., Saveliev V. & Gurevich A. MetaQUAST: evaluation of metagenome assemblies. Bioinformatics 32, 1088–1090 (2016). 26614127 10.1093/bioinformatics/btv697

[51] van der Kuyl A. C. & Cornelissen M. Identifying HIV-1 dual infections. Retrovirology 4, 67 (2007). 17892568 10.1186/1742-4690-4-67 PMC2045676

[52] Leye N. High frequency of HIV-1 infections with multiple HIV-1 strains in men having sex with men (MSM) in Senegal. Infect. Genet. Evol. J. Mol. Epidemiol. Evol. Genet. Infect. Dis. 20, 206–214 (2013). 10.1016/j.meegid.2013.09.002 24035811

[53] Baaijens J. A., Aabidine A. Z. E., Rivals E. & Schönhuth A. De novo assembly of viral quasispecies using overlap graphs. Genome Res. 27, 835–848 (2017). 28396522 10.1101/gr.215038.116 PMC5411778

[54] Chen J., Zhao Y. & Sun Y. De novo haplotype reconstruction in viral quasispecies using paired-end read guided path finding. Bioinforma. Oxf. Engl. 34, 2927–2935 (2018). 10.1093/bioinformatics/bty202 29617936

[55] Brister J. R., Ako-Adjei D., Bao Y. & Blinkova O. NCBI viral genomes resource. Nucleic Acids Res. 43, D571–577 (2015). 25428358 10.1093/nar/gku1207 PMC4383986

[56] Fritz A. CAMISIM: simulating metagenomes and microbial communities. Microbiome 7, 17 (2019). 30736849 10.1186/s40168-019-0633-6 PMC6368784

[57] Li H. The Sequence Alignment/Map format and SAMtools. Bioinforma. Oxf. Engl. 25, 2078–2079 (2009). 10.1093/bioinformatics/btp352 PMC2723002 19505943

[58] Ke Z. & Vikalo H. A Graph Auto-Encoder for Haplotype Assembly and Viral Quasispecies Reconstruction. Proc. AAAI Conf. Artif. Intell. 34, 719–726 (2020).

[59] Hage E. Characterization of Human Cytomegalovirus Genome Diversity in Immunocompromised Hosts by Whole-Genome Sequencing Directly From Clinical Specimens. J. Infect. Dis. 215, 1673–1683 (2017). 28368496 10.1093/infdis/jix157

[60] Sijmons S., Van Ranst M. & Maes P. Genomic and Functional Characteristics of Human Cytomegalovirus Revealed by Next-Generation Sequencing. Viruses 6, 1049–1072 (2014). 24603756 10.3390/v6031049 PMC3970138

[61] Hunt M. IVA: accurate de novo assembly of RNA virus genomes. Bioinforma. Oxf. Engl. 31, 2374–2376 (2015). 10.1093/bioinformatics/btv120 PMC4495290 25725497

[62] Yang X. De novo assembly of highly diverse viral populations. BMC Genomics 13, 475 (2012). 22974120 10.1186/1471-2164-13-475 PMC3469330

[63] Fedonin G. G., Fantin Y. S., Favorov A. V., Shipulin G. A. & Neverov A. D. VirGenA: a reference-based assembler for variable viral genomes. Brief. Bioinform. 20, 15–25 (2017). 10.1093/bib/bbx079 PMC6488938 28968771

[64] Prabhakaran S., Rey M., Zagordi O., Beerenwinkel N. & Roth V. HIV haplotype inference using a propagating dirichlet process mixture model. IEEE/ACM Trans. Comput. Biol. Bioinform. 11, 182–191 (2014). 26355517 10.1109/TCBB.2013.145

[65] Knyazev S. CliqueSNV: An Efficient Noise Reduction Technique for Accurate Assembly of Viral Variants from NGS Data. bioRxiv 264242 (2020) doi:10.1101/264242

[66] Splettstoesser T. English: Structure of the HIV-1 genome. It has a size of roughly 10.000 base pairs and consists of nine genes, some of which are overlapping. (2014).

[67] Mikheenko A., Valin G., Prjibelski A., Saveliev V. & Gurevich A. Icarus: visualizer for de novo assembly evaluation. Bioinformatics 32, 3321–3323 (2016). 27378299 10.1093/bioinformatics/btw379

[68] Olm M. R., Brown C. T., Brooks B. & Banfield J. F. dRep: a tool for fast and accurate genomic comparisons that enables improved genome recovery from metagenomes through de-replication. ISME J. 11, 2864–2868 (2017). 28742071 10.1038/ismej.2017.126 PMC5702732

[69] Pedregosa F. Scikit-learn: Machine Learning in Python. J. Mach. Learn. Res. 12, 2825–2830 (2011).

[70] Tarjan R. E. Depth-First Search and Linear Graph Algorithms. SIAM J Comput (1972) doi:10.1137/0201010

[71] Grenfell B. T. Unifying the epidemiological and evolutionary dynamics of pathogens. Science 303, 327–332 (2004). 14726583 10.1126/science.1090727

[72] Reimering S., Muñoz S. & McHardy A. C. Phylogeographic reconstruction using air transportation data and its application to the 2009 H1N1 influenza A pandemic. PLOS Comput. Biol. 16, e1007101 (2020). 32032362 10.1371/journal.pcbi.1007101 PMC7032730

[73] Beerenwinkel N. Geno2pheno: Estimating phenotypic drug resistance from HIV-1 genotypes. Nucleic Acids Res. 31, 3850–3855 (2003). 12824435 10.1093/nar/gkg575 PMC168981

[74] Truong D. T., Tett A., Pasolli E., Huttenhower C. & Segata N. Microbial strain-level population structure and genetic diversity from metagenomes. Genome Res. 27, 626–638 (2017). 28167665 10.1101/gr.216242.116 PMC5378180

[75] Tørresen O. K. Tandem repeats lead to sequence assembly errors and impose multi-level challenges for genome and protein databases. Nucleic Acids Res. 47, 10994–11006 (2019). 31584084 10.1093/nar/gkz841 PMC6868369

[76] Guo Y. The effect of strand bias in Illumina short-read sequencing data. BMC Genomics 13, 666 (2012). 23176052 10.1186/1471-2164-13-666 PMC3532123

[77] A reference standard for genome biology. Nat. Biotechnol. 36, 1121–1121 (2018). 30520871 10.1038/nbt.4318

[78] Suárez N. M. Human Cytomegalovirus Genomes Sequenced Directly From Clinical Material: Variation, Multiple-Strain Infection, Recombination, and Gene Loss. J. Infect. Dis. 220, 781–791 (2019). 31050742 10.1093/infdis/jiz208 PMC6667795

