# Supplementary material for: Haploflow: Strain-resolved de novo assembly of viral genomes
Source: bioRxiv. 2021 Jan 26:2021.01.25.428049. Preprint. [Version 1] doi: 10.1101/2021.01.25.428049 (PMC7852260; doi:10.1101/2021.01.25.428049)
Supplement: 1 [file NIHPP2021.01.25.428049-supplement-1.pdf]

bioRxiv preprint doi: https://doi.org/10.1101/2021.01.25.428049; this version posted January 26, 2021. The copyright holder for this preprint (which was not certified by peer review) is the author/funder, who has granted bioRxiv a license to display the preprint in perpetuity. It is made available under aCC-BY 4.0 International license.

Supplement

Exemplary clarification of path finding step realized in Haploflow

In the unitig graph there are multiple paths between a source and a sink which (sans sequencing errors) correspond to the different strains present in a sample. The choice of the correct path follows the fatness algorithm described before. There is another factor though, namely the length of the fattest path, which Haploflow *also* maximises. In Figure S1 there is exactly one source, the vertex ACTA, and one sink, the vertex ATGC, but there are infinitely many paths from ACTA to ATGC, since CTAT to TCTA and TCTA to CTAT form a loop. To prevent this, Haploflow allows every edge only to be used *once* in every path finding step. This makes the particular loop in Figure S1 "resolvable", the number of paths reduces to five:

1: $ACTA \rightarrow CTAT \rightarrow TCTA \rightarrow CTAT \rightarrow ATGC$ with a fatness of 30

2: $ACTA \rightarrow CTAT \rightarrow TCTA \rightarrow CTAC \rightarrow CTAT \rightarrow ATGC$ with a fatness of 45

3: $ACTA \rightarrow CTAT \rightarrow ATGC$ with a fatness of 75

4: $ACTA \rightarrow CTAC \rightarrow CTAT \rightarrow ATGC$ with a fatness of 25

5: $ACTA \rightarrow CTAC \rightarrow CTAT \rightarrow TCTA \rightarrow CTAT \rightarrow ATGC$ with a fatness of 25

Just going by the fattest graph, path 3 would get selected, but this path is shorter than all other paths and thus only paths 2 and 5 can be selected, out of which path 2 has the higher fatness of 45 (the coverage of the first sequence). The next longest and fattest path is path 5 with a fatness of 25 (the coverage of the last sequence) and finally path 1 remains with a fatness of 30. Paths 3 and 4 do not exist at this point, since the capacity of all edges has been used.

bioRxiv preprint doi: https://doi.org/10.1101/2021.01.25.428049; this version posted January 26, 2021. The copyright holder for this preprint (which was not certified by peer review) is the author/funder, who has granted bioRxiv a license to display the preprint in perpetuity. It is made available under aCC-BY 4.0 International license.

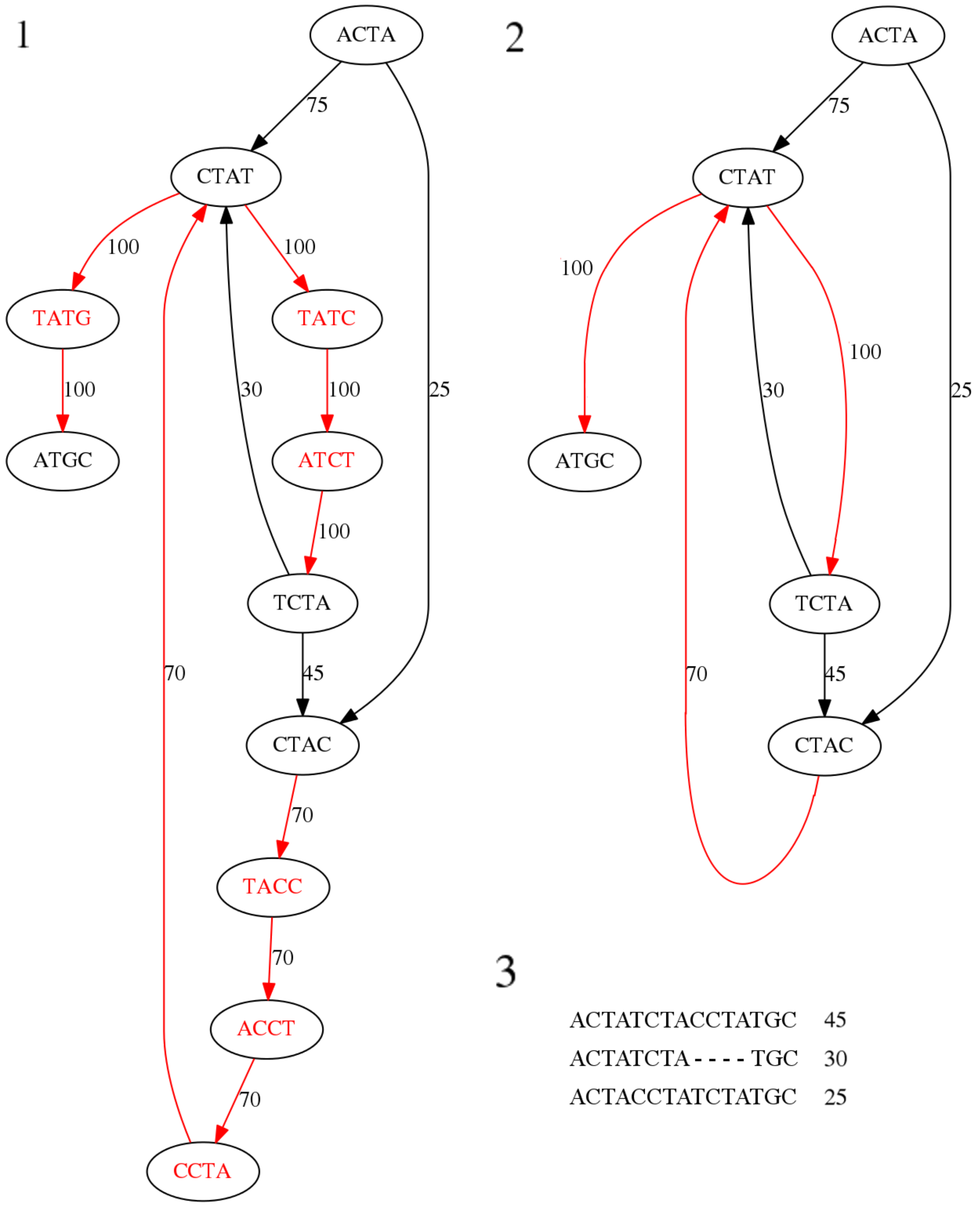

Suppl. Figure S1: The deBruijn graph (1) and its corresponding Unitig graph (2) for three related sequences and their coverage (3). The red *k*-mers and edges between them are part of linear paths and are replaced by a single red edge in the unitig graph. The edges are labelled with the "capacity", the sum of the coverages of the sequences going over them, in the deBruijn graph and the average capacity of all smoothed edges in the Unitig graph - which in

bioRxiv preprint doi: https://doi.org/10.1101/2021.01.25.428049; this version posted January 26, 2021. The copyright holder for this preprint (which was not certified by peer review) is the author/funder, who has granted bioRxiv a license to display the preprint in perpetuity. It is made available under aCC-BY 4.0 International license.

this case is the same as the original capacity. Some of the edges represent one (capacities 25, 30, 45), some two (capacity 70 = 45 + 25) and some all (capacity 100 = 45 + 30 + 25) of the sequences.

Algorithmic details of the flow algorithm

The fatness of a path is defined by the lowest fatness value of any edge along this path. Since the fatness of an edge might be underestimated if the coverage dropped for edges occurring before this edge in the path, it is not sufficient to just remove the calculated fatness when reducing flow along a path. Instead, the coverage of the source is set to 0 and for every other edge on the path the flow is reduced to *max(capacity - previously_removed_flow, 0)* where *previously_removed_flow* is the flow removed from the last edge on the path. Since it is possible that edges are used multiple times, it is also possible that there are paths that have hardly any edges that are "unique" to that path. We call an edge *unique*, if it is part of exactly one path. If the fraction or length of unique edges of a path is too low, by default less than 500 bases, the path is removed for all edges on which it is not unique, to avoid overestimating the total number of paths in the graph. Edges with coverage of 0 will get removed, possibly producing new sources. If Haploflow crosses a junction with two or more outgoing edges with similar coverage values and cannot make an informed decision which is the higher abundant path, Haploflow will break the contig at this position. This happens either if multiple strains have very similar coverages or on genomic repeats. The exact threshold for this break is derived using the *error_rate* and *strict/threshold* parameter: If the difference is less than the percentage value given or the (either explicitly stated or derived from *strict*) threshold, the contig is broken.

After the path has been found, the coverage of all unique edges on this path is reduced to 0, as no other path will be traversing this edge. If there is more than one path going over the edge, then the flow is reduced, corresponding to the expected coverage of the current edge. This value is the flow removed from the last visited unique edge, meaning that local increases and decreases in coverage are also captured. If the coverage of an edge would be reduced to 0, even though there are still paths going over this edge, the coverage is set to a dummy value such that it can still be used. On the other hand, if a path consists solely of non-unique edges, a duplication is assumed and the current path is not considered.

bioRxiv preprint doi: https://doi.org/10.1101/2021.01.25.428049; this version posted January 26, 2021. The copyright holder for this preprint (which was not certified by peer review) is the author/funder, who has granted bioRxiv a license to display the preprint in perpetuity. It is made available under aCC-BY 4.0 International license.

When permanently reducing the flow, it is not sufficient to remove the (overall) fatness of the path, since the fatness can only decrease (or stay the same) along a path, while the coverage values might fluctuate, based on amplification and sequencing strategy. To circumvent this, the flow is reduced by a "local fatness": All unique edges are removed as described before, for all other edges either flow removed from the last edge or, if the value is higher, of the average per-base removed flow, is taken as a baseline and depending on the fact whether the flow decreased or increased within the last edge, the flow to be removed is decreased and increased accordingly. If there would not be any flow remaining, a minimal value is left over.

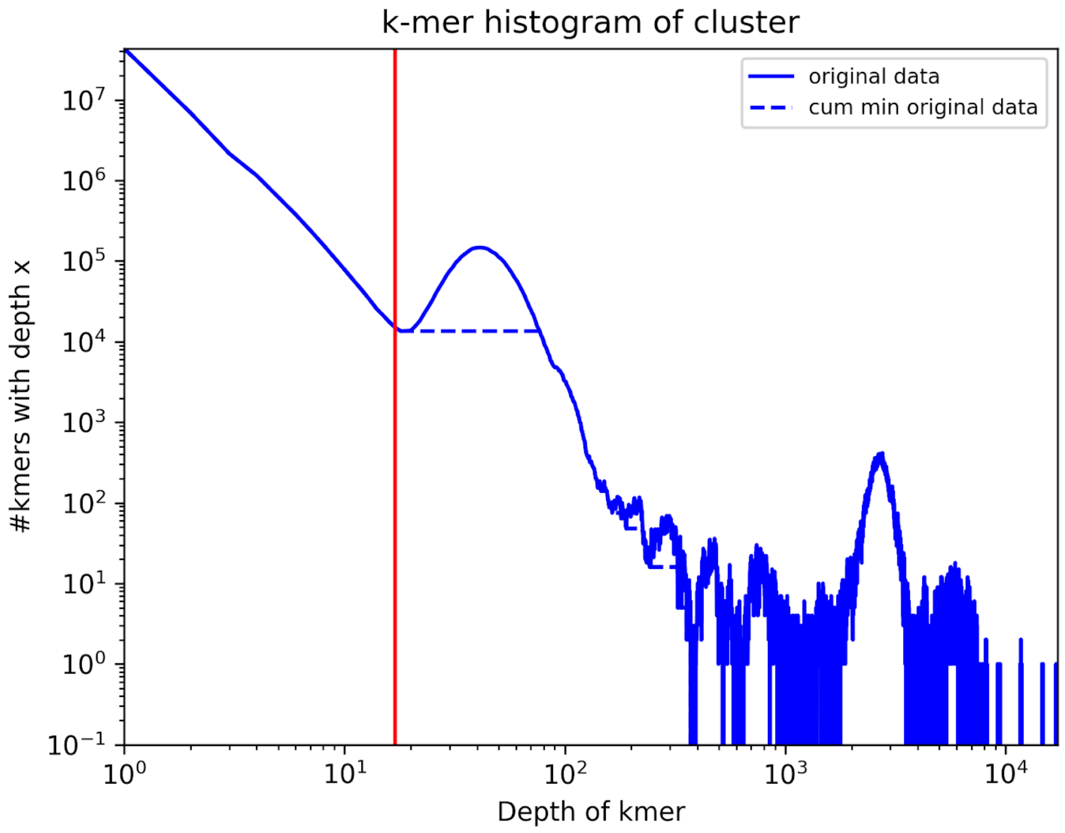

Suppl. Figure S2: log-log kmer coverage histogram for a sequence sample of HCMV strain TB40E and *E. coli*. This shows the kmer coverage (or counts) on the x-axis versus the number of kmers with that coverage on the y-axis. Original values are shown with the solid line, the cumulative minimum of the number of k-mers of a certain depth with the dashed line. k-mers with a depth less than the depth of the first k-mer for which the cumulative minimum (cum min) is less than the original value are regarded as probable erroneous k-mers (red line). For example, for a mixture of *Escherichia coli* (5,129,110 bp) and HCMV

bioRxiv preprint doi: https://doi.org/10.1101/2021.01.25.428049; this version posted January 26, 2021. The copyright holder for this preprint (which was not certified by peer review) is the author/funder, who has granted bioRxiv a license to display the preprint in perpetuity. It is made available under aCC-BY 4.0 International license.

(234,127 bp), with a length ratio of 22:1, distinct peaks occur at coverages of ~45 and ~2500. The first peak has 10,000 distinct kmers and the second one 400, indicating that the first genome might be around 25x as large as the second one.

bioRxiv preprint doi: https://doi.org/10.1101/2021.01.25.428049; this version posted January 26, 2021. The copyright holder for this preprint (which was not certified by peer review) is the author/funder, who has granted bioRxiv a license to display the preprint in perpetuity. It is made available under aCC-BY 4.0 International license.

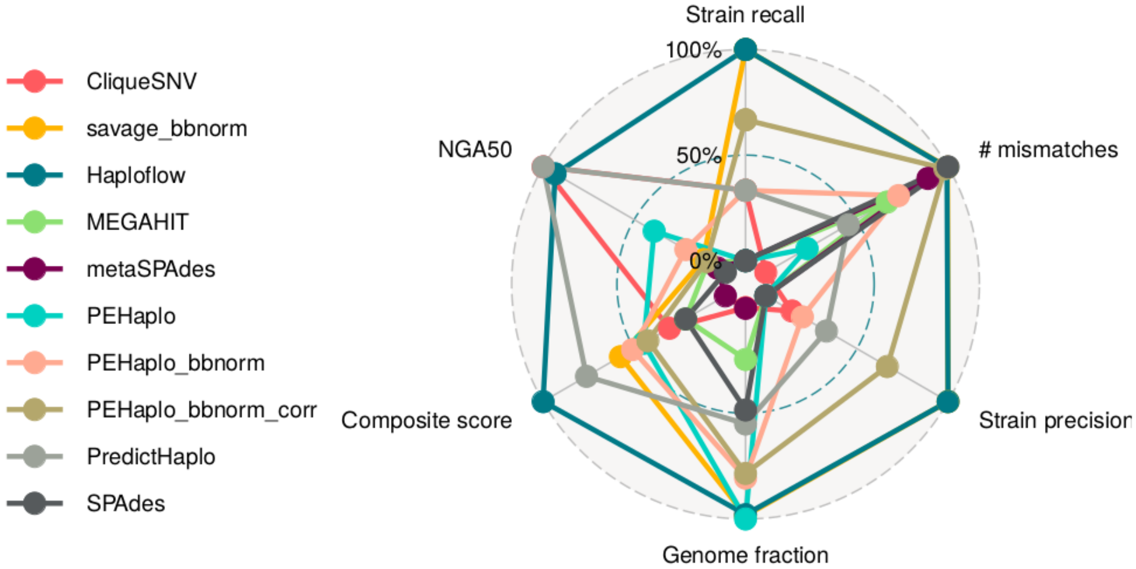

Suppl. Figure S3: Radar plot of relative performance for Haploflow and nine other methods for the HIV-3 *in silico* data set. Best performance is at 100% and Haploflow, in dark blue, ranks first in Strain recall, Strain precision and composite score.

bioRxiv preprint doi: https://doi.org/10.1101/2021.01.25.428049; this version posted January 26, 2021. The copyright holder for this preprint (which was not certified by peer review) is the author/funder, who has granted bioRxiv a license to display the preprint in perpetuity. It is made available under aCC-BY 4.0 International license.

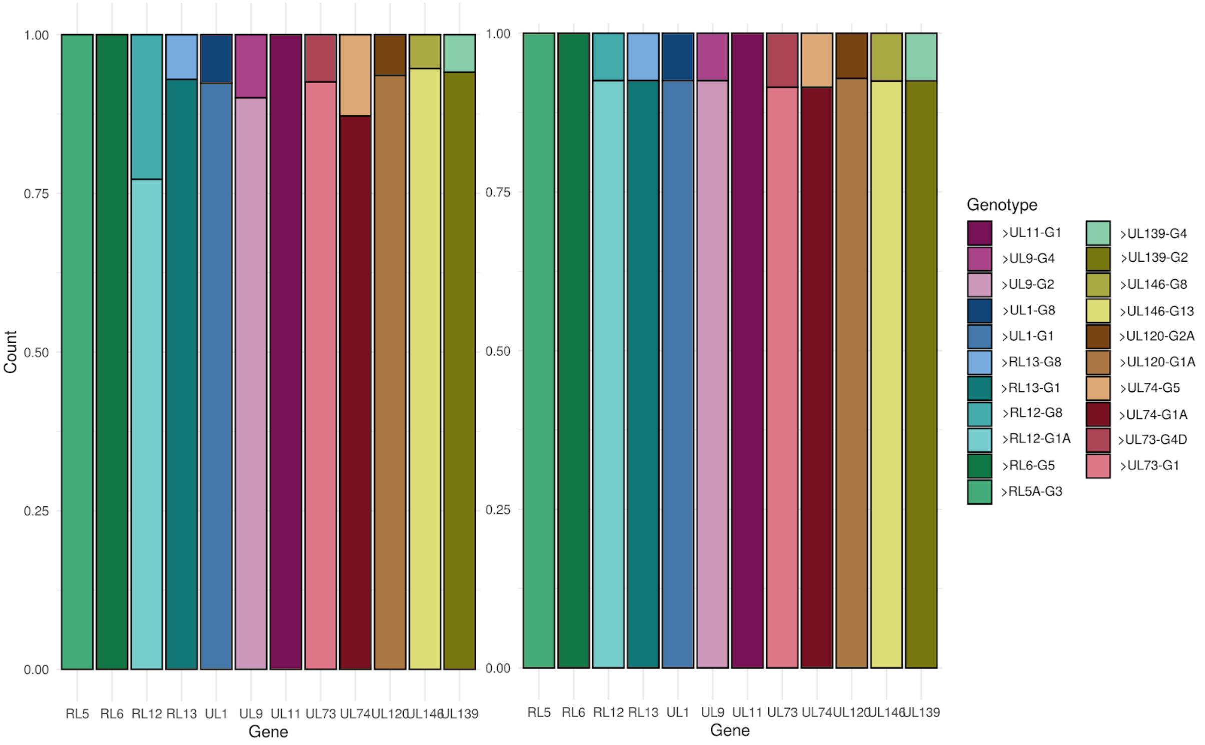

Suppl. Figure S4: Genes with different genotypes and their coverage distribution in the reads (left) and in the contigs (right). VATK was run on the reads and the contigs. For the contigs the bar height was set based on the coverage value Haploflow reports. VATK found the exact same genotypes in the reads and the Haploflow contigs.

Reconstruction of full length SARS-CoV-2 sequences

In nine out of 17 SARS-CoV-2 samples and 6 out of 7 wastewater SARS-CoV-2 samples, quast reported a high duplication ratio for the Haploflow assembly; four out of five DUS and five out of twelve WIS samples. This can be explained by either artificially duplicating parts of the genome or the presence of two closely related strains. Since Haploflow did not construct single contig assemblies for all these strains, first a “scaffolding” step was performed: All contigs are clustered using *k*-means clustering on Haploflow’s predicted abundance, the number of clusters depending on the duplication ratio. Then, using the NC_0455122.2 RefSeq strain, the contigs are extended to complete genomes, using the contigs bases for all parts of the genome covered by it. If a part of a genome is not covered by a strain, the bases from the highest abundant strain with bases at this position are inserted, if

bioRxiv preprint doi: https://doi.org/10.1101/2021.01.25.428049; this version posted January 26, 2021. The copyright holder for this preprint (which was not certified by peer review) is the author/funder, who has granted bioRxiv a license to display the preprint in perpetuity. It is made available under aCC-BY 4.0 International license.

no strain has bases at this position, the reference base is inserted. If a position is covered twice, the base from the contig with higher flow is chosen. To reduce the number of false positive SNPs, an additional filtering step was performed to remove typical sequencing and PCR related artifacts, such as deletions within homopolymeric sites[35], mutations in short-tandem repeats[75] and mutations on sites with strong strand bias[76]. This for example removed a SNP included in the original submission of the DUS sample (in the HCMV data set) at position 4655 due to a high strand bias value.

Lofreq version 2.1.4 was run on the original reads and the variants filtered by an abundance value over 5% and a score of >1000 to reduce the number of false positive calls. This filtered similar SNPs as the filtering of homopolymeric or strand biased sites performed for Haploflow.

bioRxiv preprint doi: https://doi.org/10.1101/2021.01.25.428049; this version posted January 26, 2021. The copyright holder for this preprint (which was not certified by peer review) is the author/funder, who has granted bioRxiv a license to display the preprint in perpetuity. It is made available under aCC-BY 4.0 International license.

Supplementary tables

Suppl. Table S1: Benchmark of Haploflow against five *de novo* assemblers and five reference-based assemblers (grey background) on the HIV-3 data set. For every metric, best performing methods (95-100% range of results) are indicated. *Strain recall*: fraction of correctly recovered high quality strain genomes (≤1 (≤5) mismatches per kb; more than 90% (80%) genome fraction[77]); *Strain precision*: fraction of correctly recovered high quality strain genomes of all genome assemblies. Evaluation using metaQUAST results and derived strain assembly metrics with HIV reference genomes 89.6, HXB-2 and JR-SCF in "combined reference" mode. [a]Results for a 140 Mb subset of the 500 Mb dataset generated with *BBnorm.* *runs that did not complete after ten days or failed. **as being an outlier, QuasiRecomb results were excluded from composite score and radar plot calculation.

	**Strain recall**	**Strain precision**	**Compo-site score**	**Genome fraction (%)**	**Number of contigs**	**Mis-matches**	**Dupli-cation ratio**	**NGA50**
Haploflow	**3/3**	**3/3**	**9.66**	**93.36**	**3**	**9**	**1.001**	**9083**
metaSPAdes	0/3	0/3	2.78	33.70	9	62	**1.005**	1246
SPAdes	0/3	0/3	4.28	63.09	20	**1**	**1.021**	864
MEGAHIT	0/3	0/3	4.28	48.47	12	257	**1.084**	1701
PEHaplo *l=40*	0/3	0/9	5.86	**94.63**	40	1208	3.106	4305
PEHaplo[a]	0(1)/3	1/5	6.28	82.80	20	363	1.683	2774
PEHaplo[a] *correct*	0(2)/3	2/3	5.7	81.45	17	**24**	**1.044**	1773
SAVAGE *de novo**	-	-	-	-	-	-	-	-
SAVAGE *ref**	-	-	-	-	-	-	-	-
SAVAGE[a] *ref*	*2(3)/3*	*3/3*	6.74	*93.72*	18	*5*	*1.057*	1932
PredictHaplo	1/3	1/3	8.02	67.12	3	631	1.497	*9658*
QuasiRecomb **	0/3	0/3,507	-	67.12	3506	1,011,054	1748.6	*9649*
QuasiRecomb *conservative* **	0/3	0/1,155	-	67.12	1154	411,576	575.67	*9654*

bioRxiv preprint doi: https://doi.org/10.1101/2021.01.25.428049; this version posted January 26, 2021. The copyright holder for this preprint (which was not certified by peer review) is the author/funder, who has granted bioRxiv a license to display the preprint in perpetuity. It is made available under aCC-BY 4.0 International license.

CliqueSNV	1/3	1/7	4.89	33.5	7	538	7.000	*9669*
GAEseq*	-	-	-	-	-	-	-	-
GAEseq^{a}*	-	-	-	-	-	-	-	-

Suppl. Table S2: Benchmark results for the HCMV data set. Shown are average values for the metaQUAST metrics over the six data sets and additional assembly metrics (see “performance evaluation”). For every metric, the best performing methods (95-100% range of results) are indicated. **Strain recall* includes correctly recovered genomes at two quality levels: more than 80(90)% genome fraction and less than 5(1) mismatches/kb. PEHaplo did not assemble one (TA-1-10) of the six mixtures.

	**Strain recall***	**Strain precision**	**Composite score**	**Genome fraction**	**Contigs**	**Mis-matches per 100kb**	**Dupli-cation ratio**	**NGA50**
Haploflow	**10(3)/12**	**10/14**	**9.34**	**83.87**± **10.37%**	20.50± 7.20	166.26± 122.97	1.20± 0.15	**62,560.42 ±35,233**
metaSPAdes	5(4)/12	5/12	8.18	58.57± 4.44%	17.42± 8.38	184.13± 337.14	**1.01**± **0.01**	**60,008.25 ±37,089**
SPAdes	6(4)/12	6/12	5.22	65.52± 3.94%	85.17± 15.75	40.38± 29.84	1.05± 0.01	2,552.42 ±951
MEGAHIT	2(0)/12	2/23	4.71	68.01± 8.23%	324.83± 244.29	2254.09 ±1901.7	1.92± 0.67	32,446.08 ±35,925
PEHaplo	4(3)/12	4/12	5.70	52.72± 18.07%	54.0± 78.17	**13.04**± **11.68**	1.05± 0.07	10,960.1 ±6,602
tadpole	1(0)/12	1/12	3.15	24.47± 13.31%	39.92± 12.98	27.14± 50.67	**1.00**± **0.00**	1,344.3± 3,292
ABySS	6(3)/12	6/12	6.41	64.88± 4.94%	20.92± 8.85	250.0± 85.39	1.05± 0.01	12,399.25 ±5,157
Ray	4(4)/12	4/12	5.90	51.39± 2.27%	16.67± 12.53	67.54± 63.39	1.07± 0.06	26,154.75 ±36,557
IDBA	0(0)/12	0/12	3.10	32.71± 9.52%	83.75± 13.24	104.46± 76.39	**1.03**± **0.01**	154.67± 178
Vicuna	4(1)/12	4/12	4.18	47.26± 0.93%	36.33± 7.79	104.05± 71.31	**1.02**± **0.01**	2,657.67 ±704

bioRxiv preprint doi: https://doi.org/10.1101/2021.01.25.428049; this version posted January 26, 2021. The copyright holder for this preprint (which was not certified by peer review) is the author/funder, who has granted bioRxiv a license to display the preprint in perpetuity. It is made available under aCC-BY 4.0 International license.

IVA	4(3)/12	4/12	7.42	43.23 ± 16.2%	**11.92 ± 8.22**	121.61 ± 185.89	**1.02 ± 0.03**	**63,773.0 ± 49,449**
VirGenA	*6(5)/12*	6/12	7.63	47.25 ± 1.35%	*5.67 ± 2.48*	102.41 ± 107.01	*1.01 ± 0.00*	33,324.58 ± 30,049
SAVAGE	*9(5)/12*	9/17	4.61	*82.43 ± 15.69%*	283.17 ± 98.89	33.86 ± 28.37	1.46 ± 0.14	1,245.33 ± 349

bioRxiv preprint doi: https://doi.org/10.1101/2021.01.25.428049; this version posted January 26, 2021. The copyright holder for this preprint (which was not certified by peer review) is the author/funder, who has granted bioRxiv a license to display the preprint in perpetuity. It is made available under aCC-BY 4.0 International license.

Suppl. Table S3: Genome fraction and NGA50 and their standard deviation for the high and low abundant strains in the HCMV in-vitro mixtures (two 1:10 and two 1:50 mixtures). PEHaplo and SAVAGE as reference-free haplotype assemblers did not return any results on this data set.

	metaSPAdes	MEGAHIT	Haploflow
Genome fraction (lower abundance)	19.77 ± 6.44%	30.00 ± 12.6%	**76.67 ± 12.24%**
Genome fraction (higher abundance)	**94.86 ± 0.57%**	89.97 ± 8.59%	91.62 ± 5.38%
NGA50 (lower abundance)	0 ± 0	0 ± 0	**9,625 ± 6,578**
NGA50 (higher abundance)	**149,712 ± 48,744**	45,481 ± 45,766	79,284 ± 50,679

bioRxiv preprint doi: https://doi.org/10.1101/2021.01.25.428049; this version posted January 26, 2021. The copyright holder for this preprint (which was not certified by peer review) is the author/funder, who has granted bioRxiv a license to display the preprint in perpetuity. It is made available under aCC-BY 4.0 International license.

Suppl. Table S4: One cluster with closely 11 related phage strains from the simulated virome[48] and the genome within-cluster similarities. Columns give the maximal or average ANI to all other sequences in the cluster.

GI number	Scientific name	Within-cluster similarity max	Within-cluster similarity avg
118725053	Staphylococcus phage phiNM3	98.10%	93.32%
119443652	Staphylococcus phage phiPVL108	98.31%	94.59%
157102936	Staphylococcus prophage tp310-1	99.12%	95.47%
157102938	Staphylococcus prophage tp310-3	98.74%	95.72%
239507361	Staphylococcus phage phiPVL-CN125	98.31%	94.59%
257136356	Staphylococcus phage P954	96.12%	92.18%
29028667	Staphylococcus prophage phi 13	98.74%	95.73%
30043925	Staphylococcus prophage phiN315	98.10%	92.98%
41189515	Staphylococcus phage 77	96.12%	93.13%
9635165	Staphylococcus phage PVL	99.12%	95.25%
9635677	Staphylococcus prophage phiPV83	96.59%	93.86%

bioRxiv preprint doi: https://doi.org/10.1101/2021.01.25.428049; this version posted January 26, 2021. The copyright holder for this preprint (which was not certified by peer review) is the author/funder, who has granted bioRxiv a license to display the preprint in perpetuity. It is made available under aCC-BY 4.0 International license.

Suppl. Table S5: Genome fractions in % on different subsets of the simulated virome. Unique genomes refers to genomes for which no other genome with an ANI of >95% is in the data set, common strain genomes are ones for which at least one such genome is present. The coverage value was calculated by dividing the total number of base pairs in the reads belonging to the genome by its size.

	SPAdes	MEGAHIT	Haploflow
Common strains	55.58	48.88	**62.85**
Common strains, coverage > 8	74.58	64.99	**89.36**
Total	**72.2**	68.6	66.6
Total, coverage > 8	93.07	87.55	**94.52**

bioRxiv preprint doi: https://doi.org/10.1101/2021.01.25.428049; this version posted January 26, 2021. The copyright holder for this preprint (which was not certified by peer review) is the author/funder, who has granted bioRxiv a license to display the preprint in perpetuity. It is made available under aCC-BY 4.0 International license.

Suppl. Table S6: Comparison of multiple strain infection labeling of samples by VATK[78], the predicted relative abundance of the low abundant strain(s) and the predicted abundance by Haploflow (relative and absolute) as well as the genome completeness (genome fraction, mapped against the first sample consensus genome) of strains Haploflow reconstructed (Supplementary methods). A ”-” denotes that no evidence of a second strain was found by either VATK (column 3) or Haploflow (column 4). Percentage values with a (*) denote problems in clustering, evident by a still high duplication ratio after clustering or the sum of genome fractions of two clusters summing up to ~1, indicating that underclustering or in the latter case overclustering took place. Three percentage values in the third column indicate that Haploflow predicted three strains being present.

Patient	Time points	Estimated low strain abundance (number of predicted strains)	Haploflow low strain abundance predictions (% and absolute value(s))	Genome fraction (portion of recovered genome) of strains vs. consensus sequence
RTR3	367 days	38.3% (2)	27.1% (43:16)	88.29% / 40.70%
	408 days	17.9% (2)	17.3% (167:35)	92.93% / 68.76%
RTR6	vitreous humour	9.4% (2)	12.7% (2576:373)	90.07% / 31.06%
	blood	17.3% (2)	18.4% (213:48)	99.07% / 92.73%
SCTR1	91 days	4.0% (2)	- (425)	99.69%
	126 days	22.9% (2)	21.2% (2429:653)	96.42% / 75.93%
	130 days	14.0% (2)	12.6% (313:45)	99.63% / 82.87%
	194 days	6.8% (2)	- (105)	99.78%
	224 days	37.8% (2)	26.8% (60:22)	92.73% / 79.36%
	231 days	25.4% (2)	23.6% (126:39)	92.09% / 37.63%
	244 days	- (1)	17.3% (81:17)	93.19%
	245 days	29.8% (2)	- (22)	92.86%
SCTR3	189 days	- % (1)	- (9)	98.74%
	272 days	20.5% (2)	24.3% (2781:893)	80.89% / 34.96%
	320 days	28.1% (2)	- (38)	98.46%

bioRxiv preprint doi: https://doi.org/10.1101/2021.01.25.428049; this version posted January 26, 2021. The copyright holder for this preprint (which was not certified by peer review) is the author/funder, who has granted bioRxiv a license to display the preprint in perpetuity. It is made available under aCC-BY 4.0 International license.

SCTR8	55 days	24.4% (2)	7.3% (178:14)	99.28% / 24.28%
	287 days	16.5% (2)	30.2%/8.3% (305:150:41)	93.86% / 58.5% / 52.33% (*)
SCTR11	88 days	15.6% (2)	16.3% (103:20)	99.07% / 85.76%
	192 days	11.6% (2)	11.2% (119:15)	91.75% / 78.20 %
SCTR17	21 days	34.7% (2)	22.1% (106:30)	94.81% / 30.96%
	28 days	30.6% (2)	17.6% (404:86)	98.39% / 17.04% (*)
	35 days	33.0% (2)	29.8%/9.5% (362:178:57)	99.46% / 28.08% / 22.61% (*)
	50 days	6.1% (2)	8.5% (4063:376)	97.26% / 94.18%
SCTR18	28 days	30.6% (3)	26.8% (101:37)	99.27% / 50.60% (*)
	35 days	30.3%/10.3% (3)	32.6%/10.2% (291:166:52)	80.79% / 81.88% / 48.85%
Summary strain detection		Total strains: 48	Recall: 91.7% (44/48)<br>Precision: 93.6% (44/47)	

bioRxiv preprint doi: https://doi.org/10.1101/2021.01.25.428049; this version posted January 26, 2021. The copyright holder for this preprint (which was not certified by peer review) is the author/funder, who has granted bioRxiv a license to display the preprint in perpetuity. It is made available under aCC-BY 4.0 International license.

Suppl. Table S7: SNPs and short indels detected by Haploflow and Lofreq for all 17 samples of SARS-CoV-2 in at least one sample. Lofreq was run with default parameters and SNPs were filtered by a score of >1000 and abundance >5%. “Rare” indicates thatLofreq predicted this variant at less than 5%, homopolymeric means that this site is located within or in the direct vicinity of a 4bp or longer homopolymer.

Position	Original base	Detected base	Detected by	Notes
518-520	ATG	---	Haploflow	
686-694	AAGTCATTT	---------	Haploflow	
1440	G	A	Haploflow, Lofreq	
2891	G	A	Haploflow	
4802	G	A	Lofreq	homopolymeric
7717	T	A	Haploflow, Lofreq	
10507	C	T	Haploflow, Lofreq	
11335	G	T	Haploflow, Lofreq	
11454	C	T	Haploflow, Lofreq	
11467	G	T	Haploflow, Lofreq	
11514	C	T	Haploflow, Lofreq	
11897	C	A	Haploflow, Lofreq	
12071	G	A	Haploflow	rare
13115	C	T	Haploflow, Lofreq	
15139	A	C	Haploflow, Lofreq	
15157	C	A	Lofreq	Strand bias, called by Haploflow then filtered
15168	G	A	Haploflow, Lofreq	homopolymeric
16954	C	T	Haploflow, Lofreq	
18110	C	A	Haploflow	rare
17373	C	T	Haploflow, Lofreq	

bioRxiv preprint doi: https://doi.org/10.1101/2021.01.25.428049; this version posted January 26, 2021. The copyright holder for this preprint (which was not certified by peer review) is the author/funder, who has granted bioRxiv a license to display the preprint in perpetuity. It is made available under aCC-BY 4.0 International license.

19182	A	G	Haploflow, Lofreq	
19610	C	T	Haploflow, Lofreq	
20298-20300	ATT	---	Haploflow	
21077	C	T	Lofreq	homopolymeric
21575	C	T	Haploflow	homopolymeric
22323	C	T	Haploflow, Lofreq	
25658	C	T	Haploflow, Lofreq	
29659	C	T	Lofreq	homopolymeric
29760	T	C	Lofreq	

bioRxiv preprint doi: https://doi.org/10.1101/2021.01.25.428049; this version posted January 26, 2021. The copyright holder for this preprint (which was not certified by peer review) is the author/funder, who has granted bioRxiv a license to display the preprint in perpetuity. It is made available under aCC-BY 4.0 International license.

Suppl. Table S8: Number of SARS-Cov-2 genomes assembled by Haploflow from seven SARS-CoV-2 wastewater metagenome samples and GISAID IDs of identical genomes recovered from clinical isolates. Strains are listed in order of their estimated abundances for individual samples.

Sample	# strains Haploflow	GISAID matches to strains
Oakland 5/19	2	hCoV-19/USA/LA-SR0328/2020<br>hCoV-19/USA/CA-CSMC67/2020
Oakland 5/19 (2)	2	hCoV-19/Poland/PL_P31/2020<br>hCoV-19/Beijing/DT-BJ01/2020
Oakland 5/28	2	hCoV-19/USA/CA-CSMC25/2020<br>hCoV-19/USA/CA-CSMC67/2020
Oakland 6/09	1	hCoV-19/France/IDF-10064DR/2020
Oakland 6/30	2	hCoV-19/USA/WA-UW-11903/2020<br>hCoV-19/France/IDF-10064DR/2020
Oakland 6/30 (2)	2	hCoV-19/USA/CA-CSMC25/2020<br>hCoV-19/USA/LA-SR0328/2020
Marin 7/1	2	hCoV-19/USA/VA-DCLS-1271/2020<br>hCoV-19/USA/WA-UW-11903/2020

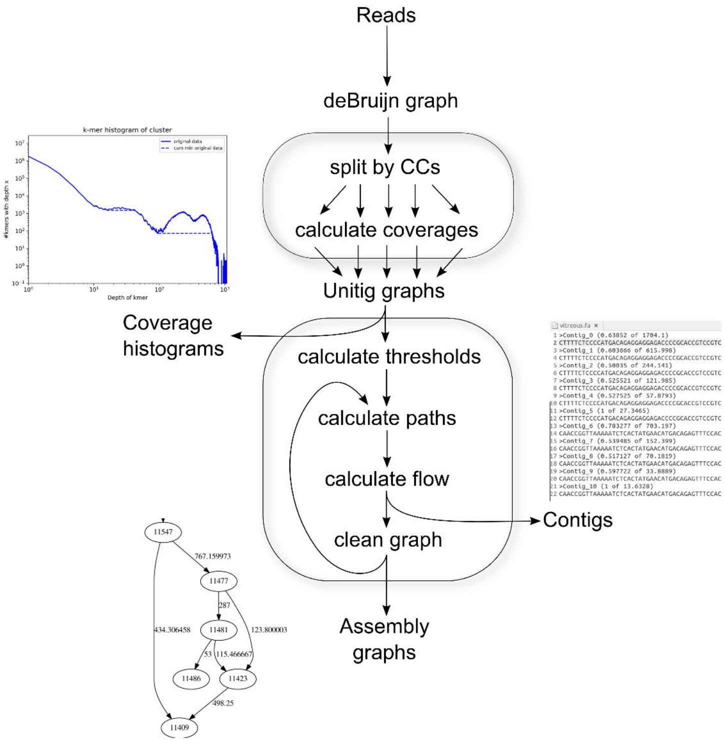

Reads
deBruijn graph
split by CCs
calculate coverages
Unitig graphs
Coverage histograms
calculate thresholds
calculate paths
calculate flow
clean graph
Contigs
Assembly graphs
k-mer histogram of cluster
original data
cum min original data
#kmers with depth x
Depth of kmer
vitreous.fa
1 >Contig_0 (0.63852 of 1704.1)
2 CTTTTCTCCCCATGACAGAGGAGGAGACCCCGCACCGTCCGTC
3 >Contig_1 (0.603666 of 615.998)
4 CTTTTCTCCCCATGACAGAGGAGGAGACCCCGCACCGTCCGTC
5 >Contig_2 (0.58035 of 244.141)
6 CTTTTCTCCCCATGACAGAGGAGGAGACCCCGCACCGTCCGTC
7 >Contig_3 (0.525521 of 121.985)
8 CTTTTCTCCCCATGACAGAGGAGGAGACCCCGCACCGTCCGTC
9 >Contig_4 (0.527525 of 57.8793)
10 CTTTTCTCCCCATGACAGAGGAGGAGACCCCGCACCGTCCGTC
11 >Contig_5 (1 of 27.3465)
12 CTTTTCTCCCCATGACAGAGGAGGAGACCCCGCACCGTCCGTC
13 >Contig_6 (0.783277 of 703.197)
14 CAACCGGTTAAAAATCTCACTATGAACATGACAGAGTTTCCAC
15 >Contig_7 (0.539485 of 152.399)
16 CAACCGGTTAAAAATCTCACTATGAACATGACAGAGTTTCCAC
17 >Contig_8 (0.517127 of 70.1819)
18 CAACCGGTTAAAAATCTCACTATGAACATGACAGAGTTTCCAC
19 >Contig_9 (0.597722 of 33.8889)
20 CAACCGGTTAAAAATCTCACTATGAACATGACAGAGTTTCCAC
21 >Contig_10 (1 of 13.6328)
22 CAACCGGTTAAAAATCTCACTATGAACATGACAGAGTTTCCAC
11547
767.159973
11477
287
434.306458
11481
123.800003
53
115.466667
11486
11423
498.25
11409

Algorithm 1: Fattest-path Dijkstra

Data: graph $G = (V, E)$ with edges $e = (v, w) \in E$, with $v, w \in V$, source $s \in V$, non-negative edge capacities $c(v, w)$ with $v, w \in V$

Result: Graph with edges labelled by their fatness, starting from source $s \in V$

forall the $v \in V - \{s\}$ **do**
    $v.fat = 0$ — set fatness to 0
    $s.dist = \infty$ — set distance to ∞
end
$Q = priority\_queue(V)$ — create priority queue keyed by $v.dist$
while $!(Q.isEmpty())$ **do**
    $u = argmax(Q, fat)$ — select fattest vertex u in Q
    $del(u, Q)$ — remove u from Q
    forall the $v \in V$ $s.t.$ $(u, v) \in E$ **do**
        Breadth-first search through G
        if $v.fat < min\{u.fat, c(u, v)\}$ **then**
            $v.fat = min\{u.fat, c(u, v)\}$ — update fatness
            $v.dist = u.dist + length(e)$ — set $dist$ as distance to source
            update Q with new $dist$ values — update Q
            $u.pred = v$ — set predecessor for backtracking
        end
    end
end

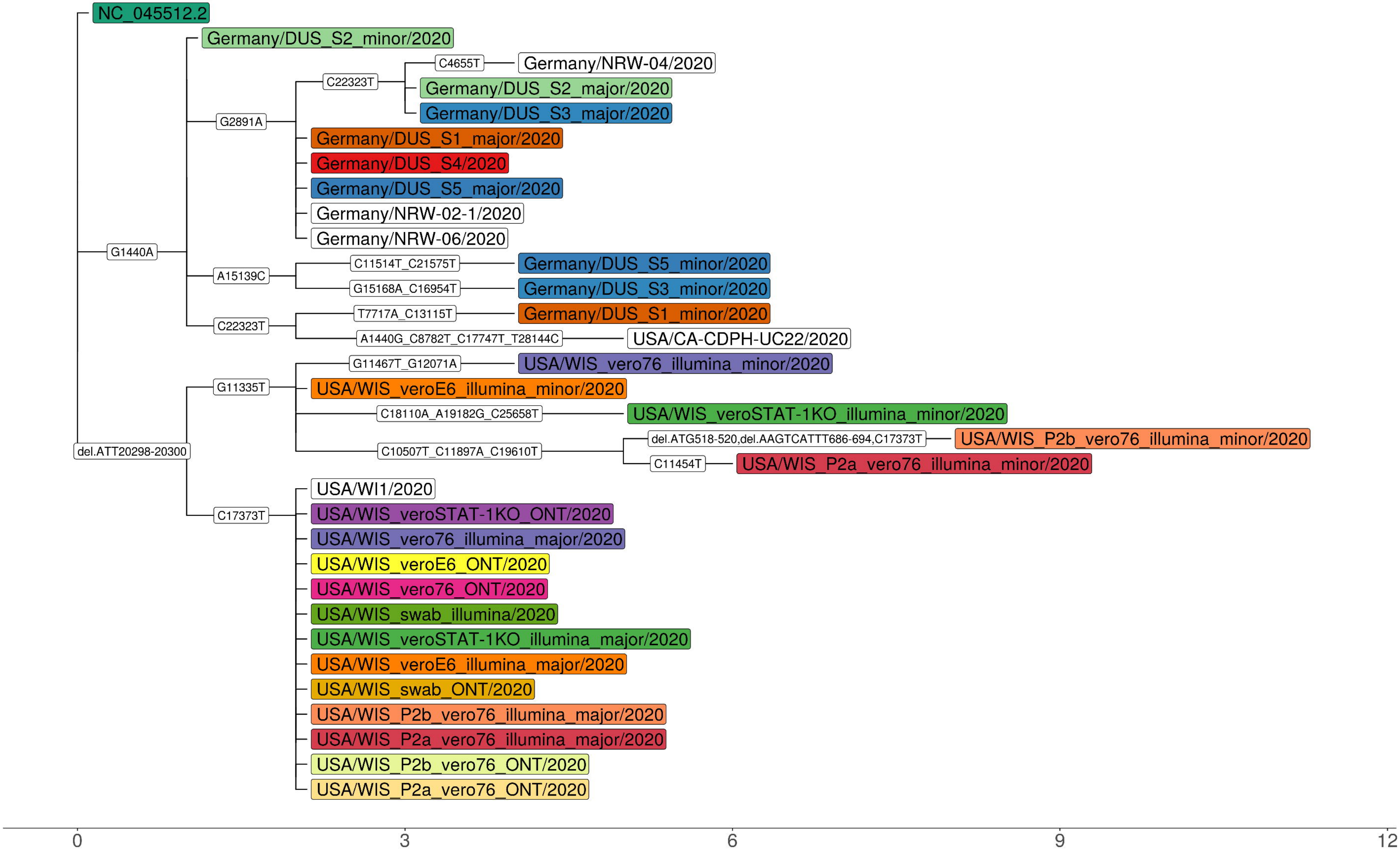

NC_045512.2
Germany/DUS_S2_minor/2020
C4655T
Germany/NRW-04/2020
C22323T
Germany/DUS_S2_major/2020
Germany/DUS_S3_major/2020
G2891A
Germany/DUS_S1_major/2020
Germany/DUS_S4/2020
Germany/DUS_S5_major/2020
Germany/NRW-02-1/2020
Germany/NRW-06/2020
G1440A
C11514T_C21575T
Germany/DUS_S5_minor/2020
A15139C
G15168A_C16954T
Germany/DUS_S3_minor/2020
T7717A_C13115T
Germany/DUS_S1_minor/2020
C22323T
A1440G_C8782T_C17747T_T28144C
USA/CA-CDPH-UC22/2020
G11467T_G12071A
USA/WIS_vero76_illumina_minor/2020
G11335T
USA/WIS_veroE6_illumina_minor/2020
C18110A_A19182G_C25658T
USA/WIS_veroSTAT-1KO_illumina_minor/2020
del.ATG518-520,del.AAGTCATTT686-694,C17373T
USA/WIS_P2b_vero76_illumina_minor/2020
del.ATT20298-20300
C10507T_C11897A_C19610T
C11454T
USA/WIS_P2a_vero76_illumina_minor/2020
USA/WI1/2020
USA/WIS_veroSTAT-1KO_ONT/2020
USA/WIS_vero76_illumina_major/2020
C17373T
USA/WIS_veroE6_ONT/2020
USA/WIS_vero76_ONT/2020
USA/WIS_swab_illumina/2020
USA/WIS_veroSTAT-1KO_illumina_major/2020
USA/WIS_veroE6_illumina_major/2020
USA/WIS_swab_ONT/2020
USA/WIS_P2b_vero76_illumina_major/2020
USA/WIS_P2a_vero76_illumina_major/2020
USA/WIS_P2b_vero76_ONT/2020
USA/WIS_P2a_vero76_ONT/2020
0
3
6
9
12

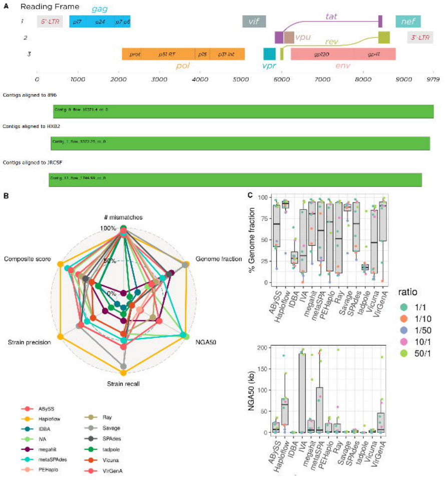

A
Reading Frame
gag
p17 p24 p7 p6
5'-LTR
vif
tat
nef
vpu
3'-LTR
rev
prot p51 RT p15 p31 int
pol
vpr
gp120 gp41
env
Contigs aligned to 896
Contigs aligned to HXB2
Contigs aligned to JRCSF
B
mismatches
Genome fraction
NGA50
Strain recall
Strain precision
Composite score
ABySS
Haploflow
IDBA
IVA
megahit
metaSPAdes
PEHaplo
Ray
Savage
SPAdes
tadpole
Vicuna
VirGenA
C
% Genome fraction
NGA50 (kb)
ratio
1/1
1/10
1/50
10/1
50/1

bioRxiv preprint doi: https://doi.org/10.1101/2021.01.25.428049; this version posted January 26, 2021. The copyright holder for this preprint (which was not certified by peer review) is the author/funder, who has granted bioRxiv a license to display the preprint in perpetuity. It is made available under aCC-BY 4.0 International license.

**Software/ Dataset**	**HIV 3 in silico mixture**		**HCMV in vitro mixture**		**Simulated virome**	
Metric	CPU *user* time (seconds)	Memory peak (GB)	Avg. CPU user time (seconds)	Avg. memory peak (GB)	CPU user time (seconds)	Memory peak (GB)
Haploflow	724	**0.009**	5,170	17.509	18,245	47.678
SAVAGE	110,208	102.938	75,518	17.658	-	-
PEHaplo	10,127	11.819	58,920	13.998	-	-
metaSPAdes	1,500	1.054	42,906	65.641	25,996	23.399
MEGAHIT	**250**	0.269	**2,910**	**0.754**	**9,690**	**2.148**

1

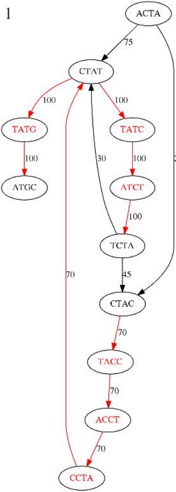

2

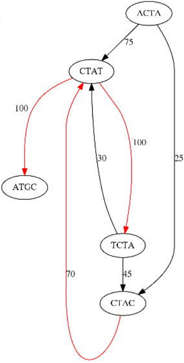

3

ACTATCTACCTATGC	45
ACTATCTA - - - - TGC	30
ACTACCTATCTATGC	25

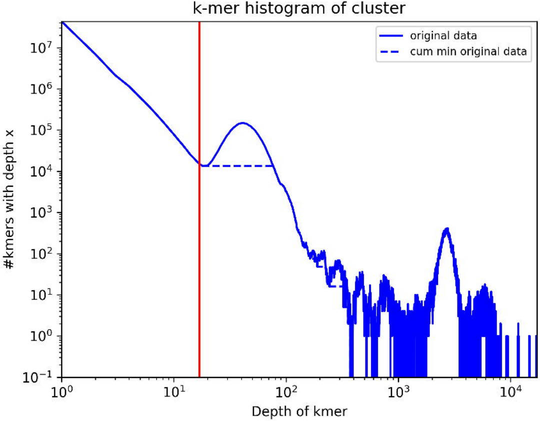

k-mer histogram of cluster
original data
cum min original data
#kmers with depth x
Depth of kmer
10^{7}
10^{6}
10^{5}
10^{4}
10^{3}
10^{2}
10^{1}
10^{0}
10^{-1}
10^{0}
10^{1}
10^{2}
10^{3}
10^{4}

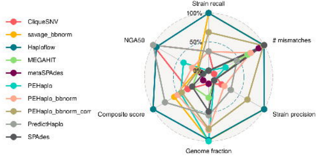

CliqueSNV
savage_bbnorm
Haploflow
MEGAHIT
metaSPAdes
PEHaplo
PEHaplo_bbnorm
PEHaplo_bbnorm_com
PredictHaplo
SPAdes
Strain recall
100%
50%
0%
mismatches
NGA50
Strain precision
Composite score
Genome fraction

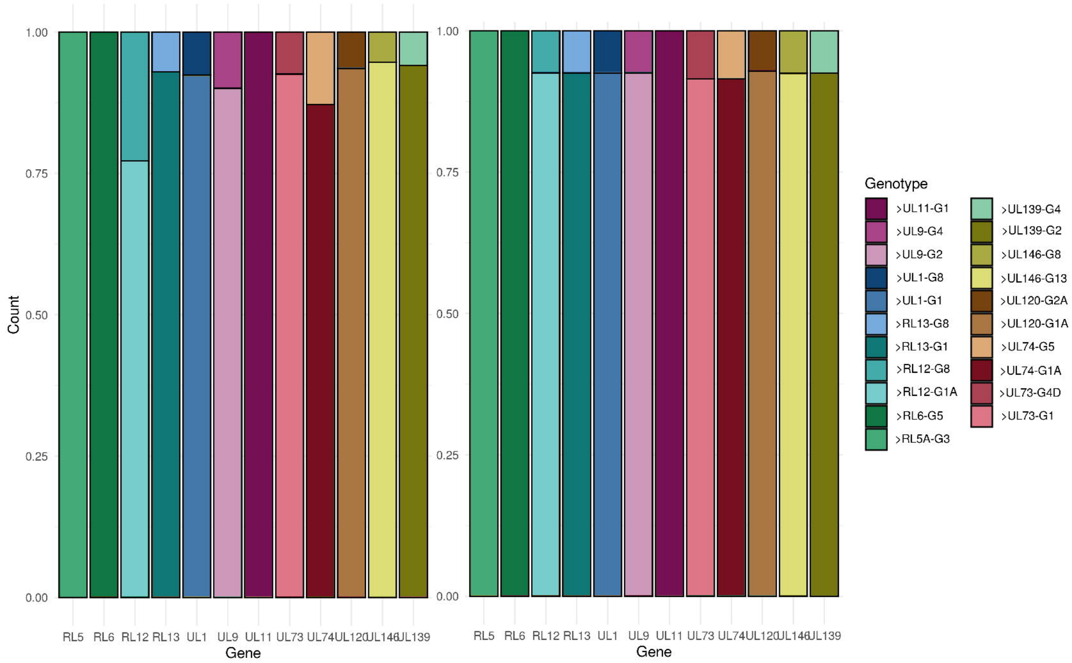
Count
1.00
0.75
0.50
0.25
0.00
RL5
RL6
RL12
RL13
UL1
UL9
UL11
UL73
UL74
UL120
UL146
UL139
Gene
Genotype
>UL11-G1
>UL9-G4
>UL9-G2
>UL1-G8
>UL1-G1
>RL13-G8
>RL13-G1
>RL12-G8
>RL12-G1A
>RL6-G5
>RL5A-G3
>UL139-G4
>UL139-G2
>UL146-G8
>UL146-G13
>UL120-G2A
>UL120-G1A
>UL74-G5
>UL74-G1A
>UL73-G4D
>UL73-G1

bioRxiv preprint doi: https://doi.org/10.1101/2021.01.25.428049; this version posted January 26, 2021. The copyright holder for this preprint (which was not certified by peer review) is the author/funder, who has granted bioRxiv a license to display the preprint in perpetuity. It is made available under aCC-BY 4.0 International license.

	**Strain recall**	**Strain precision**	**Composite score**	**Genome fraction (%)**	**Number of contigs**	**Mis-matches**	**Duplication ratio**	**NGA50**
Haploflow	**3/3**	**3/3**	**9.66**	**93.36**	**3**	**9**	**1.001**	**9083**
metaSPAdes	0/3	0/3	2.78	33.70	9	62	**1.005**	1246
SPAdes	0/3	0/3	4.28	63.09	20	**1**	**1.021**	864
MEGAHIT	0/3	0/3	4.28	48.47	12	257	**1.084**	1701
PEHaplo *l=40*	0/3	0/9	5.86	**94.63**	40	1208	3.106	4305
PEHaplo[a]	0(1)/3	1/5	6.28	82.80	20	363	1.683	2774
PEHaplo[a] *correct*	0(2)/3	2/3	5.7	81.45	17	**24**	**1.044**	1773
SAVAGE *de novo**	-	-	-	-	-	-	-	-
SAVAGE *ref* *	-	-	-	-	-	-	-	-
SAVAGE[a] *ref*	*2(3)/3*	*3/3*	6.74	*93.72*	18	*5*	*1.057*	1932
PredictHaplo	1/3	1/3	8.02	67.12	3	631	1.497	*9658*
QuasiRecomb **	0/3	0/3,507	-	67.12	3506	1,011,054	1748.6	*9649*
QuasiRecomb *conservative* **	0/3	0/1,155	-	67.12	1154	411,576	575.67	*9654*
CliqueSNV	1/3	1/7	4.89	33.5	7	538	7.000	*9669*
GAEseq*	-	-	-	-	-	-	-	-
GAEseq[a]*	-	-	-	-	-	-	-	-

bioRxiv preprint doi: https://doi.org/10.1101/2021.01.25.428049; this version posted January 26, 2021. The copyright holder for this preprint (which was not certified by peer review) is the author/funder, who has granted bioRxiv a license to display the preprint in perpetuity. It is made available under aCC-BY 4.0 International license.

	**Strain recall***	**Strain precision**	**Compo-site score**	**Genome fraction**	**Contigs**	**Mis-matches per 100kb**	**Duplication ratio**	**NGA50**
Haploflow	**10(3)/12**	**10/14**	**9.34**	**83.87± 10.37%**	20.50± 7.20	166.26± 122.97	1.20± 0.15	**62,560.42 ±35,233**
metaSPAdes	5(4)/12	5/12	8.18	58.57± 4.44%	17.42± 8.38	184.13± 337.14	**1.01± 0.01**	**60,008.25 ±37,089**
SPAdes	6(4)/12	6/12	5.22	65.52± 3.94%	85.17± 15.75	40.38± 29.84	1.05± 0.01	2,552.42 ±951
MEGAHIT	2(0)/12	2/23	4.71	68.01± 8.23%	324.83± 244.29	2254.09± 1901.7	1.92± 0.67	32,446.08 ±35,925
PEHaplo	4(3)/12	4/12	5.70	52.72± 18.07%	54.0± 78.17	**13.04± 11.68**	1.05± 0.07	10,960.1 ±6,602
tadpole	1(0)/12	1/12	3.15	24.47± 13.31%	39.92± 12.98	27.14± 50.67	**1.00± 0.00**	1,344.3± 3,292
ABySS	6(3)/12	6/12	6.41	64.88± 4.94%	20.92± 8.85	250.0± 85.39	1.05± 0.01	12,399.25 ±5,157
Ray	4(4)/12	4/12	5.90	51.39± 2.27%	16.67± 12.53	67.54± 63.39	1.07± 0.06	26,154.75 ±36,557
IDBA	0(0)/12	0/12	3.10	32.71± 9.52%	83.75± 13.24	104.46± 76.39	**1.03± 0.01**	154.67±1 78
Vicuna	4(1)/12	4/12	4.18	47.26± 0.93%	36.33± 7.79	104.05± 71.31	**1.02± 0.01**	2,657.67 ±704
IVA	4(3)/12	4/12	7.42	43.23± 16.2%	**11.92± 8.22**	121.61± 185.89	**1.02± 0.03**	**63,773.0 ±49,449**
VirGenA	*6(5)/12*	6/12	7.63	47.25± 1.35%	*5.67± 2.48*	102.41± 107.01	*1.01± 0.00*	33,324.58 ±30,049
SAVAGE	*9(5)/12*	9/17	4.61	*82.43± 15.69%*	283.17± 98.89	33.86± 28.37	1.46± 0.14	1,245.33 ± 349

bioRxiv preprint doi: https://doi.org/10.1101/2021.01.25.428049; this version posted January 26, 2021. The copyright holder for this preprint (which was not certified by peer review) is the author/funder, who has granted bioRxiv a license to display the preprint in perpetuity. It is made available under aCC-BY 4.0 International license.

	metaSPAdes	MEGAHIT	Haploflow
Genome fraction (lower abundance)	19.77±6.44%	30.00±12.6%	**76.67±12.24%**
Genome fraction (higher abundance)	**94.86±0.57%**	89.97±8.59%	91.62±5.38%
NGA50 (lower abundance)	0±0	0±0	**9,625±6,578**
NGA50 (higher abundance)	**149,712±48,744**	45,481±45,766	79,284±50,679

bioRxiv preprint doi: https://doi.org/10.1101/2021.01.25.428049; this version posted January 26, 2021. The copyright holder for this preprint (which was not certified by peer review) is the author/funder, who has granted bioRxiv a license to display the preprint in perpetuity. It is made available under aCC-BY 4.0 International license.

GI number	Scientific name	Within-cluster similarity max	Within-cluster similarity avg
118725053	Staphylococcus phage phiNM3	98.10%	93.32%
119443652	Staphylococcus phage phiPVL108	98.31%	94.59%
157102936	Staphylococcus prophage tp310-1	99.12%	95.47%
157102938	Staphylococcus prophage tp310-3	98.74%	95.72%
239507361	Staphylococcus phage phiPVL-CN125	98.31%	94.59%
257136356	Staphylococcus phage P954	96.12%	92.18%
29028667	Staphylococcus prophage phi 13	98.74%	95.73%
30043925	Staphylococcus prophage phiN315	98.10%	92.98%
41189515	Staphylococcus phage 77	96.12%	93.13%
9635165	Staphylococcus phage PVL	99.12%	95.25%
9635677	Staphylococcus prophage phiPV83	96.59%	93.86%

bioRxiv preprint doi: https://doi.org/10.1101/2021.01.25.428049; this version posted January 26, 2021. The copyright holder for this preprint (which was not certified by peer review) is the author/funder, who has granted bioRxiv a license to display the preprint in perpetuity. It is made available under aCC-BY 4.0 International license.

	SPAdes	MEGAHIT	Haploflow
Common strains	55.58	48.88	**62.85**
Common strains, coverage > 8	74.58	64.99	**89.36**
Total	**72.2**	68.6	66.6
Total, coverage > 8	93.07	87.55	**94.52**

bioRxiv preprint doi: https://doi.org/10.1101/2021.01.25.428049; this version posted January 26, 2021. The copyright holder for this preprint (which was not certified by peer review) is the author/funder, who has granted bioRxiv a license to display the preprint in perpetuity. It is made available under aCC-BY 4.0 International license.

Patient	Time points	Estimated low strain abundance (number of predicted strains)	Haploflow low strain abundance predictions (% and absolute value(s))	Genome fraction (portion of recovered genome) of strains vs. consensus sequence
RTR3	367 days	38.3% (2)	27.1% (43:16)	88.29% / 40.70%
	408 days	17.9% (2)	17.3% (167:35)	92.93% / 68.76%
RTR6	vitreous humour	9.4% (2)	12.7% (2576:373)	90.07% / 31.06%
	blood	17.3% (2)	18.4% (213:48)	99.07% / 92.73%
SCTR1	91 days	4.0% (2)	- (425)	99.69%
	126 days	22.9% (2)	21.2% (2429:653)	96.42% / 75.93%
	130 days	14.0% (2)	12.6% (313:45)	99.63% / 82.87%
	194 days	6.8% (2)	- (105)	99.78%
	224 days	37.8% (2)	26.8% (60:22)	92.73% / 79.36%
	231 days	25.4% (2)	23.6% (126:39)	92.09% / 37.63%
	244 days	- (1)	17.3% (81:17)	93.19%
	245 days	29.8% (2)	- (22)	92.86%
SCTR3	189 days	- % (1)	- (9)	98.74%
	272 days	20.5% (2)	24.3% (2781:893)	80.89% / 34.96%
	320 days	28.1% (2)	- (38)	98.46%
SCTR8	55 days	24.4% (2)	7.3% (178:14)	99.28% / 24.28%
	287 days	16.5% (2)	30.2%/8.3% (305:150:41)	93.86% / 58.5% / 52.33% (*)
SCTR11	88 days	15.6% (2)	16.3% (103:20)	99.07% / 85.76%
	192 days	11.6% (2)	11.2% (119:15)	91.75% / 78.20 %

bioRxiv preprint doi: https://doi.org/10.1101/2021.01.25.428049; this version posted January 26, 2021. The copyright holder for this preprint (which was not certified by peer review) is the author/funder, who has granted bioRxiv a license to display the preprint in perpetuity. It is made available under aCC-BY 4.0 International license.

SCTR17	21 days	34.7% (2)	22.1% (106:30)	94.81% / 30.96%
	28 days	30.6% (2)	17.6% (404:86)	98.39% / 17.04% (*)
	35 days	33.0% (2)	29.8%/9.5% (362:178:57)	99.46% / 28.08% / 22.61% (*)
	50 days	6.1% (2)	8.5% (4063:376)	97.26% / 94.18%
SCTR18	28 days	30.6% (3)	26.8% (101:37)	99.27% / 50.60% (*)
	35 days	30.3%/10.3% (3)	32.6%/10.2% (291:166:52)	80.79% / 81.88% / 48.85%
Summary strain detection		Total strains: 48	Recall: 91.7% (44/48)<br>Precision: 93.6% (44/47)	

bioRxiv preprint doi: https://doi.org/10.1101/2021.01.25.428049; this version posted January 26, 2021. The copyright holder for this preprint (which was not certified by peer review) is the author/funder, who has granted bioRxiv a license to display the preprint in perpetuity. It is made available under aCC-BY 4.0 International license.

Position	Original base	Detected base	Detected by	Notes
518-520	ATG	---	Haploflow	
686-694	AAGTCATT T	---------	Haploflow	
1440	G	A	Haploflow, Lofreq	
2891	G	A	Haploflow	
4802	G	A	Lofreq	homopolymeric
7717	T	A	Haploflow, Lofreq	
10507	C	T	Haploflow, Lofreq	
11335	G	T	Haploflow, Lofreq	
11454	C	T	Haploflow, Lofreq	
11467	G	T	Haploflow, Lofreq	
11514	C	T	Haploflow, Lofreq	
11897	C	A	Haploflow, Lofreq	
12071	G	A	Haploflow	rare
13115	C	T	Haploflow, Lofreq	
15139	A	C	Haploflow, Lofreq	
15157	C	A	Lofreq	Strand bias, called by Haploflow then filtered
15168	G	A	Haploflow, Lofreq	homopolymeric
16954	C	T	Haploflow, Lofreq	
18110	C	A	Haploflow	rare
17373	C	T	Haploflow, Lofreq	
19182	A	G	Haploflow, Lofreq	
19610	C	T	Haploflow, Lofreq	
20298-20300	ATT	---	Haploflow	
21077	C	T	Lofreq	homopolymeric

bioRxiv preprint doi: https://doi.org/10.1101/2021.01.25.428049; this version posted January 26, 2021. The copyright holder for this preprint (which was not certified by peer review) is the author/funder, who has granted bioRxiv a license to display the preprint in perpetuity. It is made available under aCC-BY 4.0 International license.

21575	C	T	Haploflow	homopolymeric
22323	C	T	Haploflow, Lofreq	
25658	C	T	Haploflow, Lofreq	
29659	C	T	Lofreq	homopolymeric
29760	T	C	Lofreq	

bioRxiv preprint doi: https://doi.org/10.1101/2021.01.25.428049; this version posted January 26, 2021. The copyright holder for this preprint (which was not certified by peer review) is the author/funder, who has granted bioRxiv a license to display the preprint in perpetuity. It is made available under aCC-BY 4.0 International license.

Sample	# strains Haploflow	GISAID matches to strains
Oakland 5/19	2	hCoV-19/USA/LA-SR0328/2020<br>hCoV-19/USA/CA-CSMC67/2020
Oakland 5/19 (2)	2	hCoV-19/Poland/PL_P31/2020<br>hCoV-19/Beijing/DT-BJ01/2020
Oakland 5/28	2	hCoV-19/USA/CA-CSMC25/2020<br>hCoV-19/USA/CA-CSMC67/2020
Oakland 6/09	1	hCoV-19/France/IDF-10064DR/2020
Oakland 6/30	2	hCoV-19/USA/WA-UW-11903/2020<br>hCoV-19/France/IDF-10064DR/2020
Oakland 6/30 (2)	2	hCoV-19/USA/CA-CSMC25/2020<br>hCoV-19/USA/LA-SR0328/2020
Marin 7/1	2	hCoV-19/USA/VA-DCLS-1271/2020<br>hCoV-19/USA/WA-UW-11903/2020
